# Genome Analysis of a Verrucomicrobial Endosymbiont With a Tiny Genome Discovered in an Antarctic Lake

**DOI:** 10.3389/fmicb.2021.674758

**Published:** 2021-06-01

**Authors:** Timothy J. Williams, Michelle A. Allen, Natalia Ivanova, Marcel Huntemann, Sabrina Haque, Alyce M. Hancock, Sarah Brazendale, Ricardo Cavicchioli

**Affiliations:** ^1^School of Biotechnology and Biomolecular Sciences, UNSW Sydney, Sydney, NSW, Australia; ^2^U.S. Department of Energy Joint Genome Institute, Berkeley, CA, United States

**Keywords:** Antarctic microbiology, Bacterial endosymbionts, metagenome, extreme genome reduction, genetic code 4

## Abstract

Organic Lake in Antarctica is a marine-derived, cold (−13∘C), stratified (oxic-anoxic), hypersaline (>200 gl^–1^) system with unusual chemistry (very high levels of dimethylsulfide) that supports the growth of phylogenetically and metabolically diverse microorganisms. Symbionts are not well characterized in Antarctica. However, unicellular eukaryotes are often present in Antarctic lakes and theoretically could harbor endosymbionts. Here, we describe *Candidatus* Organicella extenuata, a member of the Verrucomicrobia with a highly reduced genome, recovered as a metagenome-assembled genome with genetic code 4 (UGA-to-Trp recoding) from Organic Lake. It is closely related to *Candidatus* Pinguicocccus supinus (163,218 bp, 205 genes), a newly described cytoplasmic endosymbiont of the freshwater ciliate *Euplotes vanleeuwenhoeki* (Serra et al., 2020). At 158,228 bp (encoding 194 genes), the genome of *Ca.* Organicella extenuata is among the smallest known bacterial genomes and similar to the genome of *Ca.* Pinguicoccus supinus (163,218 bp, 205 genes). *Ca.* Organicella extenuata retains a capacity for replication, transcription, translation, and protein-folding while lacking any capacity for the biosynthesis of amino acids or vitamins. Notably, the endosymbiont retains a capacity for fatty acid synthesis (type II) and iron–sulfur (Fe-S) cluster assembly. Metagenomic analysis of 150 new metagenomes from Organic Lake and more than 70 other Antarctic aquatic locations revealed a strong correlation in abundance between *Ca.* Organicella extenuata and a novel ciliate of the genus *Euplotes*. Like *Ca.* Pinguicoccus supinus, we infer that *Ca.* Organicella extenuata is an endosymbiont of *Euplotes* and hypothesize that both *Ca.* Organicella extenuata and *Ca.* Pinguicocccus supinus provide fatty acids and Fe-S clusters to their *Euplotes* host as the foundation of a mutualistic symbiosis. The discovery of *Ca.* Organicella extenuata as possessing genetic code 4 illustrates that in addition to identifying endosymbionts by sequencing known symbiotic communities and searching metagenome data using reference endosymbiont genomes, the potential exists to identify novel endosymbionts by searching for unusual coding parameters.

## Introduction

Bacteria with highly reduced genome sizes are only found as host-restricted symbionts and pathogens (Supplementary Table 1; Moran and Bennett, 2014). The smallest bacterial genomes are only known to occur in symbionts that are required by a host (obligate symbionts), with those possessing genomes < 500 kbp being completely dependent on the host while also providing benefit to the host to be retained (mutualistic symbionts) (Moran and Bennett, 2014). Insects that feed on sap (phloem or xylem) rely on endosymbionts to supplement their restrictive or unbalanced diets; these bacteria, either individually or as “patchworks” of metabolically complementary co-symbionts or nested symbionts, provide essential amino acids and/or vitamins for their respective protist hosts (Nakabachi and Ishikawa, 1999; Zientz et al., 2004; Nakabachi et al., 2006; Pérez-Brocal et al., 2006; Bennett and Moran, 2013; Brown et al., 2015; Gil et al., 2018). The cellulolytic protists that reside in the hindguts of termites harbor cytoplasmic endosymbionts that belong to diverse bacterial clades (e.g., Endomicrobia, Deltaproteobacteria, Bacteroidetes, and Actinobacteria) and confer metabolic and nutritional benefits to their respective protist hosts (Stingl et al., 2005; Ohkuma et al., 2007; Hongoh et al., 2008a, b; Sato et al., 2009; Ikeda-Ohtsubo et al., 2016; Strassert et al., 2016; Kuwahara et al., 2017).

Verrucomicrobia is a diverse phylum of bacteria that has been found in a wide array of habitats, with free-living representatives isolated from soils, seawater, marine sediments, lakes, and hot springs (Wagner and Horn, 2006; Dunfield et al., 2007; Yoon et al., 2007a,b). Certain verrucomicrobia live in close association with eukaryotes, including marine sponges (Scheuermayer et al., 2006; Yoon et al., 2008) and tunicates (Lopera et al., 2017), as well as inside the intestinal tracts of humans (Derrien et al., 2004), termites (Wertz et al., 2012), and marine clam worms (Choo et al., 2007). Some verrucomicrobia have entered into very close symbiotic associations with eukaryotic hosts, including anti-predator ectosymbionts (epixenosomes) of the ciliate *Euplotidium* (Petroni et al., 2000) and various endosymbionts, such as inside the cytoplasm of intestinal and ovarial cells of nematode worms (Vandekerckhove et al., 2002), nuclei of cellulolytic protists (Sato et al., 2014), and the cytoplasm of the ciliate *Euplotes vanleeuwenhoeki* (Serra et al., 2020). *Candidatus* Xiphinematobacter, the verrucomicrobial endosymbiont of nematodes, has a 0.916-Mbp metagenome assembled genome (MAG) encoding 817 predicted protein-coding sequences (CDS); compared with free-living relatives, genes are retained for the biosynthesis of amino acids predicted to be required by their nematode hosts (Brown et al., 2015). The unpublished MAG of the intranuclear endosymbiont *Ca.* Nucleococcus (Sato et al., 2014) is ∼1 Mbp and encodes ∼700 CDS (Y. Hongoh, personal communication). The genome of the *Euplotes* endosymbiont *Ca.* Pinguicoccus supinus has an ‘extremely reduced genome’ at only 0.163 Mbp and encodes 168 CDS (Serra et al., 2020).

Organic Lake is a shallow (∼7 m deep), marine-derived, Antarctic lake formed ∼3,000 years ago (Gibson, 1999). The lake is characterized by a salinity gradient that reaches a maximum of ∼230 g L^–1^ (Gibson, 1999) and has unusual chemistry, with very high levels of dimethylsulfide (Gibson et al., 1991). Temperatures in the upper waters have been recorded as high as 15°C and as low as −14°C (Franzmann et al., 1987), whereas bottom waters (5 – ∼7 m) have typically registered temperatures of −5 to −6°C (Franzmann et al., 1987; Gibson et al., 1991; Roberts et al., 1993; James et al., 1994) but as low as −13°C (Yau et al., 2013). Metaproteogenomic analyses have inferred important roles for virophage-mediated control of algal primary production (Yau et al., 2011) and roles in nutrient cycling by phylogenetically and metabolically diverse bacteria (Yau et al., 2013). The lake is located in the Vestfold Hills, a ∼ 400 km^2^ region of East Antarctica that contains hundreds of water bodies, many of which are marine-derived, having been formed ∼3,000–7,000 years ago as a result of the isostatic rebound of the continent (Gibson, 1999; Cavicchioli, 2015; Supplementary Figure 1). The water bodies in the Vestfold Hills range in salinity from freshwater to hypersaline, most of which have not been subject to metagenomic analysis of their biota (Cavicchioli, 2015).

During analyses of unusual coding parameters (genetic code 4) in metagenome contigs, we discovered a 158-kbp verrucomicrobial MAG that was assembled from new metagenome data derived from a complete seasonal cycle of Organic Lake. The MAG is comparable in size with the endosymbiont *Ca.* Pinguicoccus supinus (Serra et al., 2020), as well as to obligate mutualistic endosymbionts that belong to the phyla Proteobacteria and Bacteroidetes that also have extremely reduced genomes (Moran and Bennett, 2014). The environmental distribution and inferred *Euplotes* host of the Organic Lake endosymbiont was assessed by analyzing 337 Antarctic metagenomes, including 150 new metagenomes of unstudied Vestfold Hills lakes and neighboring marine locations. The Organic Lake endosymbiont is closely related to *Ca.* Pinguicoccus; herein, we describe the functional traits of this bacterial lineage that seem to underpin the endosymbiosis and discuss the value of searching for unusual coding parameters as a means of identifying endosymbionts.

## Materials and Methods

### Sampling and DNA Extraction

Microbial biomass was obtained and field observations recorded from lakes in the Vestfold Hills, Antarctica (Supplementary Figure 1). Sampling at Organic Lake was performed by sequential size fractionation through a 20-μm prefilter onto 3.0-, 0.8-, and 0.1-μm large-format membrane-filters (293 mm diameter polyethersulfone), samples preserved and DNA extracted, as described previously (Yau et al., 2011, 2013; Tschitschko et al., 2018; Panwar et al., 2020).

For other lakes, including Unnamed Lake 18, “Portals” Lake, Unnamed Lake 13, Unnamed Lake 17, “Swamp” Lake, Unnamed Lake 12, and Unnamed Lake 7, biomass was captured using Sterivex cartridges (MilliporeSigma, Burlington, MA, United States) by pumping water from the lake through a 20-μm prefilter using a hand-driven peristaltic pump. After field collection, Sterivex cartridges were kept cold (e.g., in snow) before transportation to Davis Research Station, where they were cryogenically preserved at −80∘C and shipped at −80∘C to Australia. To extract DNA, the Sterivex cartridge was removed from −80∘C storage and filled with 1.6 ml of freshly prepared “XS” buffer (1% potassium ethyl xanthogenate; 100-mM Tris-hydrochloride, pH 7.4; 20-mM ethylenediamine tetraacetic acid, pH 8; 1% sodium dodecyl sulfate; 800-mM ammonium acetate) (Tillett and Neilan, 2000). Both ends of the cartridge were sealed with parafilm, and the cartridge was placed into an empty 50-ml Falcon tube and incubated in a water bath at 65°C for 2 h. After incubation, 200 μl of 10% sodium dodecyl sulfate and 50 μl of 20 mg ml^–1^ Proteinase K (Thermo Fisher Scientific, Waltham, MA, United States) was added through the Luer-lock end of the cartridge, re-sealed, and returned to the 50-ml Falcon tube for incubation in a water bath at 55°C for 2 h. After incubation, a syringe was attached to the Luer-lock end and air injected to recover the liquid in a 20-ml Falcon tube. The liquid was decanted, placing 500-μl aliquots into 1.5-ml microfuge tubes, 60 μl of phenol added, the tubes inverted several times to mix the solution, 500 μl of chloroform: isoamyl alcohol (24:1) was added, and each tube mixed by inversion. The tubes were centrifuged at 16,800 × *g* for 10 min at room temperature, the aqueous phase of each sample was collected into a fresh 1.5-ml tube, 1.5 μl of GlycoBlue (Thermo Fisher Scientific) was added to each tube, and tubes were left at room temperature for 1 h. Ammonium acetate (3 M, 500 μl) was added to each tube, mixed by inversion, left at room temperature for 30 min, tubes centrifuged at 16,800 × *g* for 15 min, and the supernatant placed into fresh 2-ml tubes. A total of 1 ml of 100% ethanol was added to each tube, and after storage overnight at 4°C, tubes were centrifuged at 14,000 × *g* for 30 min at room temperature and the supernatant carefully discarded. Pellets were washed by adding 500 μl of 70% ethanol and tubes centrifuged at 14,000 × *g* for 5 min. Ethanol was removed, the pellets air-dried on a heating block at 37°C, pellets resuspended in Tris-hydrochloride–ethylenediaminetetraacetic acid buffer (10-mM Tris-hydrochloride, pH 7.4; 1-mM ethylenediamine tetraacetic acid, pH 8) and tubes stored at −80°C. DNA yields were quantified using Qubit dsDNA BR Assay Kit (Thermo Fisher Scientific) and the quality of DNA assessed by agarose gel electrophoresis. DNA was sequenced at the Joint Genome Institute using Hi-Seq2500 (2 × 151 bp run) as described previously (Tschitschko et al., 2018; Panwar et al., 2020) or at the Australian Centre for Ecogenomics using NextSeq500 (on a 2 × 150 bp run) and raw reads filtered using Trimmomatic (Trimmomatic manual: V0.32, no date). Assembly was performed with metaSpades and all contigs > 200 bp uploaded and annotated by the IMG pipeline (Huntemann et al., 2015).

### Analyses of DNA Sequence Data

The *Ca.* Organicella MAG was identified using a pipeline to identify stop codon reassignments in metagenomic data (Ivanova et al., 2014). The set of contigs with potential UGA reassignment was identified in Organic Lake metagenomes based on the higher total coding potential as computed by Prodigal upon reannotation with genetic code 4. These contigs had an average GC content of 32%, and they appeared to have characteristics of bacterial genomes, namely, high coding density, typical bacterial gene complement with translation, transcription, and replication machinery, but no multi-subunit NADH dehydrogenase and cytochrome oxidase complexes indicative of mitochondria and no photosynthesis genes indicative of chloroplasts. The longest of these contigs, which were ∼158 kb, turned out to be circular due to an overlap of 100 nt at the ends. No other putative bacterial contigs with UGA reassignment were found in the same metagenomes, suggesting that these circular contigs constituted the entire genome of a bacterium. Because the automated annotations initially performed by IMG used genetic code 11, in which UGA is a stop codon, manual inspection of these contigs identified genes interrupted by stop codons within open reading frames. Re-calling open reading frames and annotating the genome using PROKKA (Seemann, 2014) with codon chart 4, reassigned the opal stop codon (UGA) as tryptophan. This reduced the number of genes from 249 to 193 for the reference *Ca.* Organicella extenuata MAG (contigID Ga0307966_1000010). Total coding density was calculated using all protein-coding genes (CDS), rRNA, tRNA, and tmRNA genes in the genome. Protein identities were determined using ExPASy BLAST for all CDS and, where necessary, InterProScan and HHPred. The isoelectric point (pI) of protein sequences was determined using the Isoelectric Point Calculator (Kozlowski, 2016). The genomic functional potential was assessed by considering cellular and metabolic traits based upon manual examination of genes and pathways performed in a similar way to previous assessments of the veracity of gene functional assignments (Allen et al., 2009; Panwar et al., 2020).

Mapping of reads from 340 Antarctic metagenomes to the *Ca.* Organicella MAG was performed using BWA v0.7.17 (Li and Durbin, 2009). FastANI (Jain et al., 2018) was used to calculate ANI between *Ca.* Organicella MAGs. Multiple alignments were constructed using Clustal (DNA sequences) (Thompson et al., 1994) or MUSCLE (protein sequences) (Edgar, 2004) and used to construct phylogenetic trees (for *Ca.* Organicella and for *Euplotes* sp. AntOrgLke) by the maximum-likelihood method (Tamura and Nei, 1993) in MEGA6 (Tamura et al., 2013) with 1,000 bootstraps. Marker genes predicted from the *Ca.* Organicella MAG were used to place the MAG into a concatenated 43-marker gene tree by CheckM (Parks et al., 2014) using the tree command.

To identify the potential host(s) of *Ca.* Organicella, six metagenomes where *Ca.* Organicella was abundant (Org-646, Org-46, Org-175, Org-784, Portals, and UnnamedLake18) were selected to create a co-assembly using Megahit v.1.2.2b (Li et al., 2016), with contigs binned into 188 MAGs by Metabat v.2.12.1 (Kang et al., 2019) with default settings (min contig length 2,500). During the co-assembly, a single contig representing the *Ca.* Organicella MAG was assembled (k141_311079; 158,131 bp). As this *Ca.* Organicella MAG was not initially binned, due to falling below the default minimum bin size of 200 kb, it was manually assigned to bin189. The ANI between the original *Ca.* Organicella MAG (Ga0307966_1000010) and the co-assembled MAG (k141_311079) was 99.9924%. The *Ca.* Organicella MAG (bin189) and the 188 bins resulting from Metabat binning were screened for contamination, completion, and taxonomic identity using checkM (Parks et al., 2014) and refineM (Parks et al., 2017). In addition, the abundance of each bin [calculated as the sum of (contig length × contig coverage) for all contigs in the bin] was determined for each of the 29 metagenomes where *Ca.* Organicella was detected by mapping the metagenome reads to the bins with bbmap v38.51 (Bushnell, 2014). These bin abundances were used as input for SparCC (Friedman and Alm, 2012) implemented in python 3^1^ to estimate correlation values from the compositional data.

To identify the taxonomy of the bins that were highly correlated to *Ca.* Organicella, MetaEuk v. 20200908 (Levy Karin et al., 2020) was used to identify eukaryotic proteins and assign taxonomy *via* the 2bLCA lowest common ancestor approach. To identify the maximal number of proteins, the larger MERC_MMETSP_Uniclust50_profiles database was used as the reference dataset for MetaEuk, whereas to assign contig taxonomy and putative protein function, the TaxDB_uniclust90_2018_08 database was used. Both databases were obtained from http://wwwuser.gwdg.de/∼compbiol/metaeuk/. The rRNA gene contig missing from the *Euplotes* sp. AntOrgLke MAG was identified as contig k141_859071 by blasting *Euplotes* spp. 18S rRNA genes against the co-assembled contigs used for Metabat. 18S/28S rRNA genes on k141_859071 were predicted using the RNAmmer 1.2 Server at http://www.cbs.dtu.dk/services/RNAmmer/ (Lagesen et al., 2007). The mitochondrial genome of *Euplotes* sp. AntOrgLke was identified by blasting the mitochondrial proteins of *E. vanleeuwenhoeki, Euplotes crassus*, and *Euplotes minuta* against the co-assembled contigs used for the Metabat binning, with the resulting contigs then blasted against the metagenome where *Ca*. Organicella was most abundant.

As few non-mitochondrial *Euplotes* proteins were available in the National Center for Biotechnology Information (NCBI) nr database, additional protein sequences were gathered from five reference *Euplotes* species. The data were obtained from genome-specific databases: *Euplotes octocarinatus*, http://ciliates.ihb.ac.cn/database/home/#eo (Wang et al., 2018); *Euplotes vannus*, http://evan.ciliate.org/ (Chen et al., 2019); from proteins predicted from the Marine Microbial Eukaryote Meta/transcriptome sequencing project (MMETSP): *Euplotes harpa* FSP1.4, IMG ID 3300017294; *Euplotes focardii* TN1, IMG IDs 3300017169 and 3300016941; *E. crassus* CT5, IMG ID 3300017039; all accessed at https://img.jgi.doe.gov/; by manually running MetaEuk for protein prediction on genome sequences held in NCBI Genome: *E. focardii*, GCA_001880345.1 ASM188034v1; *E. crassus* GCA_001880385.1 ASM188038v1; or by manually running MetaEuk on genomic data held in a custom database: *E. vannus*, http://evan.ciliate.org/. Average amino acid identity (AAI) was calculated at http://enve-omics.ce.gatech.edu/aai/index (Rodriguez-R and Konstantinidis, 2016) between the *Euplotes* sp. AntOrgLke and the five reference *Euplotes* species, using the protein sequences downloaded or predicted for their respective genomes (*Euplotes* sp. AntOrgLke – 15328 proteins predicted in this study; *E. octocarinatus* – 29076 proteins obtained from http://ciliates.ihb.ac.cn/database/home/#eo; *E. focardii* TN1, *E. crassus* CT5, and *E. harpa* FSP1.4 – 12634, 12729, and 19386 proteins, respectively, predicted from the Marine Microbial Eukaryote Meta/transcriptome sequencing project (MMETSP) and accessed at http://img.jgi.doe.gov; *E. vannus* – 43338 proteins obtained from http://evan.ciliate.org).

To correctly identify the CDS in the *Ca*. Pinguicoccus genome, Prokka (Seemann, 2014) was used with genetic code 4. To investigate the relationship between *Ca*. Organicella and the newly released *Ca*. Pinguicoccus genome, nucleotide synteny was visualized with progressiveMauve (Darling et al., 2004), a tblastx plot was performed at NCBI^2^, and AAI was calculated at http://enve-omics.ce.gatech.edu/aai/index (Rodriguez-R and Konstantinidis, 2016), followed by manual inspection of protein identifications to identify shared and unique metabolic capacities.

## Results and Discussion

### Organic Lake MAG Summary and Phylogeny

A MAG (Ga0307966_1000010) representing a complete circular genome with a length of 158,228 bp was identified in new metagenome data from Organic Lake. The MAG encoded 194 bacterial genes, 156 of which were inferred to be CDS (Supplementary Table 2) with 145 assigned putative biological functions (Supplementary Table 3). Most (76 proteins) were assigned to translation (including tRNA modifications) (Supplementary Table 3). Other categories were fatty acid synthesis (including pyruvate oxidation) (18 proteins); cell wall biogenesis including lipopolysaccharides (17), iron–sulfur (Fe-S) cluster assembly (8), protein folding and stability (8), replication and repair (6), and transcription (6). A total of 16 CDS could not be assigned any function, and some or all of these could be pseudogenes. The MAG had one copy each of 23S, 16S, and 5S rRNA genes and 34 identifiable tRNA genes (Supplementary Table 2). The highly restricted genomic potential illustrates this bacterium would not be capable of autonomous growth, and we name it *Candidatus* Organicella extenuata gen. et. sp. nov.; the genus name derives from the locality from where the MAG sequence was originally recovered (Organic Lake, Antarctica) with the addition of the diminutive Latin suffix -ella; the species “extenuata” means reduced or diminished in Latin and is in reference to the highly reduced genome.

**FIGURE 1:**
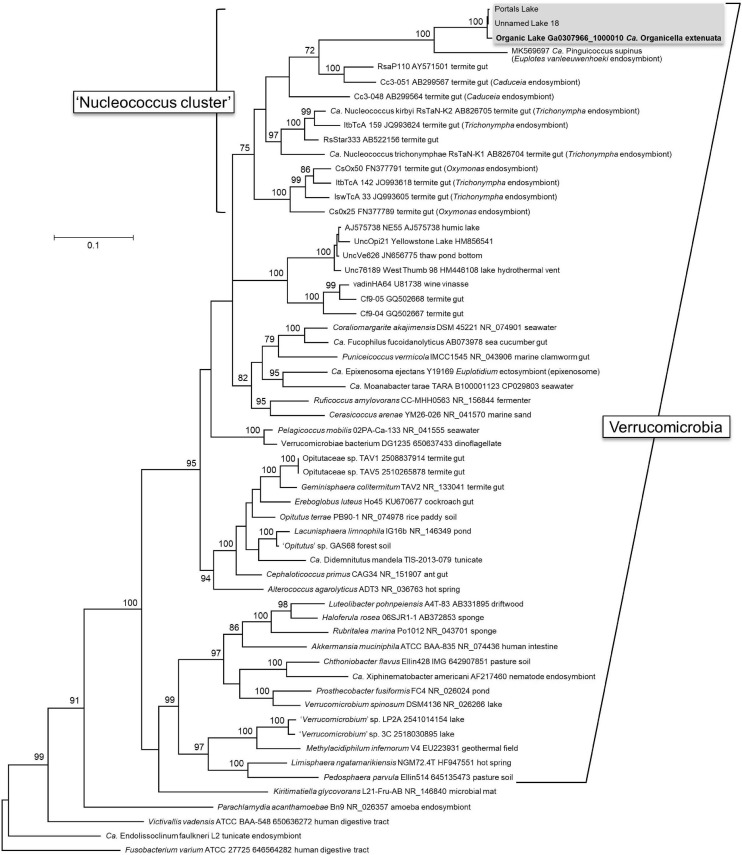
Phylogeny of *Candidatus* Organicella extenuata. Phylogeny of Verrucomicrobia and related bacteria based on 16S rRNA sequences, showing *Ca.* Organicella extenuata nested inside the newly proposed “Nucleococcus cluster”; other than *Ca.* Organicella extenuata, this cluster comprises the cytoplasmic endosymbiont *Ca.* Pinguicoccus supinus from a freshwater ciliate and intranuclear endosymbionts of amitochondriate protists resident in the hindgut of termites. The maximum likelihood tree was constructed with 59 sequences, and positions with less than 80% site coverage were eliminated, resulting in 1,415 positions in the final dataset. Bootstrap values > 70 are shown next to individual nodes. *Fusobacterium varium* is the outgroup. Accessions are given as NCBI Nucleotide accessions or IMG Gene IDs: for *Ca.* Organicella extenuata, sequences were included for the original Organic Lake MAG (contig Ga0307966_1000010, bases 107297..108828), Unnamed Lake 18 (contig Ga0400283_000007, bases 52431..53966), and “Portals” Lake (contig Ga0400669_009478, bases 1..1071 and contig Ga0400669_039189, bases 1314..1821). Sequences identical to the 16S rRNA sequence from the original Organic Lake MAG were represented in metagenome data from 19 other Organic Lake metagenomes and also in Unnamed Lake 13 (Supplementary Table 4). Note that nine-digit accessions are IMG Gene IDs, and all others are NCBI Nucleotide accessions.

Additional MAGs for *Ca.* Organicella were generated from a number of Antarctic metagenomes (see section *Ca. Organicella Environmental Distribution and Host* below), enabling the analysis of 23 *Ca.* Organicella 16S rRNA genes (Supplementary Table 4). Phylogenetic analysis of these genes found *Ca.* Organicella to be most closely related to *Ca.* Pinguicoccus (Serra et al., 2020), with 85% 16S rRNA gene identity (see section *Comparison of Ca. Organicella and Ca. Pinguicoccus Genomes* below). Both *Ca.* Organicella and *Ca.* Pinguicoccus belong to a cluster of uncultured Verrucomicrobia that also includes *Ca.* Nucleococcus and related endosymbionts of certain amitochondriate protists (*Trichonympha*, *Caduceia*, and *Oxymonas*) present in termite hindguts (Yang et al., 2005; Hongoh et al., 2007; Ikeda-Ohtsubo et al., 2010; Sato et al., 2014; Figure 1). This cluster, previously termed the “termite cluster” (Sato et al., 2014), is not closely related to other known verrucomicrobial endosymbionts (Vandekerckhove et al., 2002) or ectosymbionts (Petroni et al., 2000). In view of the cluster now including *Ca.* Organicella and *Ca.* Pinguicoccus, and no longer containing species exclusive to the termite gut, we suggest the cluster be termed the “Nucleococcus cluster.” To date, known representatives of this “Nucleococcus cluster” of Verrucomicrobia include both nuclear and cytoplasmic endosymbionts of unicellular eukaryotes.

The *Ca.* Organicella + *Ca.* Pinguicoccus branch within the “Nucleococcus cluster” of the 16S rRNA gene tree was far longer than other branches (Figure 1), and similar topology occurred in trees constructed using conserved marker genes (Supplementary Figure 2). Such long branches were not evident for any other sequences, including the endosymbionts *Ca.* Nucleococcus and *Ca.* Xiphinematobacter (Figure 1), both of which had much larger genomes (∼ 1 Mbp) than *Ca.* Organicella and *Ca.* Pinguicoccus (Supplementary Table 1). Long branches likely reflect rapid sequence evolution and are characteristic of degenerate genomes (McCutcheon and Moran, 2012), consistent with *Ca.* Organicella and *Ca.* Pinguicoccus being the only known representatives of Verrucomicrobia with extremely reduced genomes.

### Endosymbiont Features

The *Ca.* Organicella MAG exhibits a number of features typical of obligate symbionts that have highly reduced genomes (McCutcheon and Moran, 2010, 2012). The MAG has a high coding density (95% for all genes and 90% for CDS only), with shortened intergenic regions and 23 overlapping genes (Supplementary Table 1), which is characteristic of extreme genome reduction (Nakabachi et al., 2006; Moya et al., 2008). The MAG has genetic code 4^3^ with UGA stop codons recoded to tryptophan. Of note is that a tRNA-Opal-TCA is also encoded (Ga0307966_1000010189) that has the highest similarity to trnW (UGA) from mitochondria of *Paralemanea* sp. (GenBank accession MG787097.1). UGA-to-Trp recoding is known to occur rarely, having been found in mycoplasmas (Yamao et al., 1985); certain symbiotic bacteria (McCutcheon et al., 2009), including *Ca.* Pinguicoccus (Serra et al., 2020); and several mitochondrial lineages (Knight et al., 2001). The UGA-to-Trp conversion permits the loss of peptide chain release factor 2 (PrfB) (which recognizes UGA codons) through genome erosion (McCutcheon et al., 2009). UGA-to-Trp recoding is typically associated with low GC content (McCutcheon et al., 2009), although some insect endosymbionts with UGA-to-Trp have high GC content (e.g., *Ca.* Hodgkinia cicadicola 58%; *Ca.* Tremblaya princeps PCIT 59%) (McCutcheon and Moran, 2012). The GC content of the *Ca.* Organicella MAG is 32%, compared with 25% for *Ca.* Pinguicoccus (Serra et al., 2020) (also see *Comparison of Ca. Organicella and Ca. Pinguicoccus Genomes*, later). No mobile elements were identified in the *Ca.* Organicella MAG, which is another trait of symbionts with extremely reduced genomes (McCutcheon and Moran, 2012).

The possession of a minimal complement of genes required for transcription and translation (McCutcheon, 2010; McCutcheon and Moran, 2012), and some capacity to perform DNA replication, enables a level of autonomy over cellular processes that distinguishes endosymbiotic bacteria from organelles (McCutcheon and Moran, 2012). *Ca.* Organicella encodes some enzymes involved in DNA replication, including DNA gyrase (GyrAB), DNA primase (DnaG), and replicative DNA helicase (DnaB), but a dedicated DNA polymerase for DNA replication was not identifiable. Although certain insect endosymbionts lack the DNA polymerase III holoenzyme, they at least encode DNA polymerase α-subunit (DnaE), responsible for 5’ to 3’ polymerization activity of DNA replication (McCutcheon, 2010; McCutcheon and Moran, 2012). In the absence of DnaE, genomic replication is presumably carried out by host proteins (Serra et al., 2020). As in many other reduced endosymbiont genomes, *Ca.* Organicella lacks the DnaA protein for initiation of DNA replication, and this function is presumably carried out by the host (Gil et al., 2003; López-Madrigal et al., 2013), possibly as a mechanism to exercise control over endosymbiont proliferation (e.g., Akman et al., 2002; Gil et al., 2003; Bennett et al., 2014; Bennett and Moran, 2015).

Three subunits of the DNA-directed RNA polymerase (RNAP) for transcription were identified (RpoA, RpoB, and RpoC) as well as a sigma factor (RpoD), components that are typical of endosymbionts (McCutcheon and Moran, 2012). Thus, the components of RNAP retained by *Ca.* Organicella parallel those of unrelated symbionts with genomes of comparable size (McCutcheon, 2010; McCutcheon and Moran, 2012). A total of 34 amino acyl tRNAs for all 20 proteinogenic amino acids were identified, plus aminoacyl tRNA synthetases (aaRS) for 13 of the amino acids (Met, Leu, Ile, Val, Lys, Gly, Ser, Cys, Arg, Tyr, Ala, Phe, and Glu) and a glutamyl/aspartyl-tRNA amidotransferase. The missing aaRS may be provided by the host (Van Leuven et al., 2019), or existing aaRS may catalyze multiple aminoacylation reactions (Moran and Bennett, 2014). *Ca.* Organicella encodes initiation factors IF-1 and IF-2 (but not IF-3); elongation factors EF-G, EF-Ts, and EF-4; translational release factor PrfA (but not PrfB); and ribosome recycling factor. Most, but not all, ribosomal subunits were identified. Known endosymbionts with highly reduced genomes typically do not encode a complete set of ribosomal proteins (McCutcheon, 2010; Moran and Bennett, 2014). Individual ribosomal subunits that could not be identified in the *Ca.* Organicella MAG are also missing from some obligate insect endosymbionts (e.g., RplA, RpmC, RpmD, RpsF, and RpmF) (Moran and Bennett, 2014). Certain tRNA modification enzymes were also evident in the *Ca.* Organicella MAG (e.g., Mnm complex and TsaD) that are usually retained in endosymbionts (McCutcheon and Moran, 2012; Van Leuven et al., 2019) (see *Supplementary Text – tRNA Modification*).

The only identifiable dedicated DNA repair enzyme in *Ca.* Organicella was a RecA homolog. Depleted DNA repair abilities are typical of bacteria with highly reduced genomes and contribute to the accumulation of deleterious substitutions, including in CDS (McCutcheon and Moran, 2012; Bennett and Moran, 2015). The average predicted pI of *Ca.* Organicella proteins was 9.2 (Supplementary Table 3). It has been proposed that high (alkaline) pI of the proteome of intracellular parasites and endosymbionts may result from the accumulation of mutations (Kiraga et al., 2007). However, not all *Ca.* Organicella proteins were predicted to have a high pI. Notably, the two most acidic proteins are ferredoxin (pI 4.1) and acyl carrier protein (ACP) (pI 4.2), both of which are naturally acidic proteins (Knaff and Hirasawa, 1991; McAllister et al., 2006). If high pI does arise from high rates of mutation, the acidic pI of ferredoxin and ACP may be indicative of a strong positive selection to preserve function.

Another trait that is shared between *Ca.* Organicella and known bacterial symbionts with highly reduced genomes is the retention of chaperone proteins (GroES-GroEL; DnaK); these chaperone proteins are thought to ameliorate the adverse effects of accumulated deleterious substitutions on correct protein-folding (Moran, 1996; McCutcheon and Moran, 2012). The bacteria that synthesize these chaperones are therefore heat-sensitive, limiting the thermal tolerance of their hosts (Burke et al., 2010; Fan and Wernegreen, 2013; Moran and Bennett, 2014). Thermal instability would not be expected to be a problem for *Ca.* Organicella in Antarctica (Franzmann et al., 1987; Gibson, 1999; Yau et al., 2013). Proteins that are damaged and cannot be correctly re-folded could be degraded to peptides by the encoded ClpXP (Sabree et al., 2013), although the fate of the peptides is unclear in the absence of identifiable peptidases.

### *Ca.* Organicella Environmental Distribution and Host

To examine the environmental distribution of *Ca.* Organicella, 337 Antarctic lake and marine metagenomes were analyzed, which encompass 77 different Antarctic aquatic locations, including a time (December 2006 to January 2015) and depth series of Organic Lake (Supplementary Figure 1 and Supplementary Table 5). Sequence coverage of *Ca.* Organicella MAGs from Organic Lake was higher at depth in the lake and higher in winter compared with spring or summer (Supplementary Table 5). Although the highest abundance of *Ca.* Organicella was from Organic Lake (up to a median read depth of 71), read coverage showed *Ca.* Organicella was also present in seven other lakes in the Vestfold Hills (Supplementary Figure 1), including a complete MAG from a small pond ∼15 km away from Organic Lake (“Unnamed Lake 18”), which had a median read depth of 22 and coverage of the original *Ca.* Organicella MAG (Ga0307966_1000010) of 99.97% (Supplementary Table 5). The MAGs from Organic Lake (11 close to full length) had an ANI of ≥ 99.5%, with the ANI across all MAGs from Organic Lake, Unnamed Lake 18, Portals Lake, and Unnamed Lake 13, ≥98.1%. Outside of these *Ca.* Organicella MAGs and *Ca.* Pinguicoccus, the best BLAST matches to the *Ca.* Organicella 16S rRNA gene in NCBI-nr and IMG databases were ≤ 82%. This indicates that a single species of *Ca.* Organicella is present in the Vestfold Hills, with *Ca.* Pinguicoccus being the only similar species identifiable elsewhere in the world.

**FIGURE 2:**
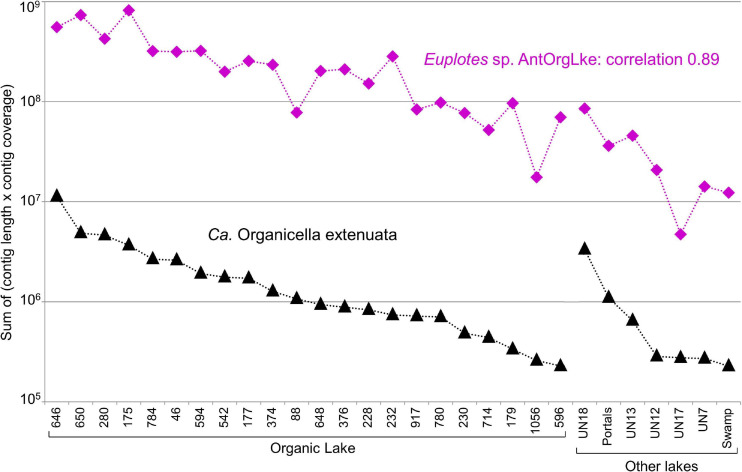
Co-occurrence of *Candidatus* Organicella extenuata and *Euplotes* sp. AntOrgLke in Antarctic metagenomes. The abundance of *Ca.* Organicella extenuata (k141_311079) and *Euplotes* sp. AntOrgLke (bin81 + bin14 + contigk141_859071), calculated as the sum of (contig length × contig coverage) for all contigs, was analyzed using SparCC to determine their co-occurrence (*r*, correlation coefficient). Across 29 metagenomes in which *Ca.* Organicella extenuata was detected, the abundance of *Euplotes* sp. AntOrgLke strongly positively correlated with the abundance of *Ca.* Organicella extenuata (*r* = 0.89, *p* = 0), indicating *Euplotes* sp. AntOrgLke was likely the host of *Ca.* Organicella extenuata. None of the other 187 bins representing other potential hosts exhibited a positive correlation above *r* = 0.54. *X*-axis labels: Organic Lake, metagenome IDs (see Supplementary Table 5); Other lakes, lake names (Unnamed abbreviated as UN).

To identify the potential host(s) of *Ca.* Organicella, metagenomes were co-assembled using Metabat, generating a *Ca.* Organicella MAG (k141_311079) plus 188 potential host bins. The abundance of each bin was determined for each of the 29 metagenomes where *Ca.* Organicella was detected by mapping the metagenome reads to the bins, and the correlation of bin abundances was calculated using SparCC. The abundance of *Ca.* Organicella was highly positively correlated with bin81 (*r* = 0.89, *p* = 0), bin149 (*r* = 0.95, *p* = 0), and contig k141_859071 (*r* = 0.85, *p* = 0). The two bins and the contig were also highly positively correlated to each other (*r* = 0.94 – 0.99, *p* = 0). Bin81 (12,580 contigs) and bin149 (18 contigs) were dominated by sequences assigned to the ciliate *Euplotes* (Euplotidae, Spirotrichea, and Ciliophora), and the 8.1-kb contig, k141_859071 contained a 28S rRNA gene (4,455 bp), region of a 5.8S rRNA gene and 18S rRNA gene (1,895 bp) that matched to *Euplotes* (e.g., 28S rRNA, 84.2% identity to *Euplotes aediculatus* across 79% of query length). We infer that bins 81 and 149 plus the rRNA contig represent a MAG that pertains to a single OTU that we refer to as “*Euplotes* sp. AntOrgLke” (Supplementary Table 6). The *Euplotes* sp. AntOrgLke MAG (Supplementary Dataset 1) comprises 29.98 Mbp across 12,599 contigs (longest contig 19,935 bp, N50 = 2,645, L50 = 3,806, GC = 38.15%), with 6,451 proteins predicted against the TaxDB_uniclust90_2018_08 database (Supplementary Dataset 2) and 15,328 proteins predicted against the MERC_MMETSP_Uniclust50_profiles database (Supplementary Dataset 3). Of relevance, the abundance of the *Ca.* Organicella MAG was highly positively correlated with the *Euplotes* sp. AntOrgLke MAG (*r* = 0.89, *p* = 0) (Figure 2), consistent with this ciliate being the host. Moreover, contigs belonging to the *Euplotes* sp. AntOrgLke mitochondrial genome were also detected (Supplementary Table 7; Supplementary Dataset 4).

**FIGURE 3:**
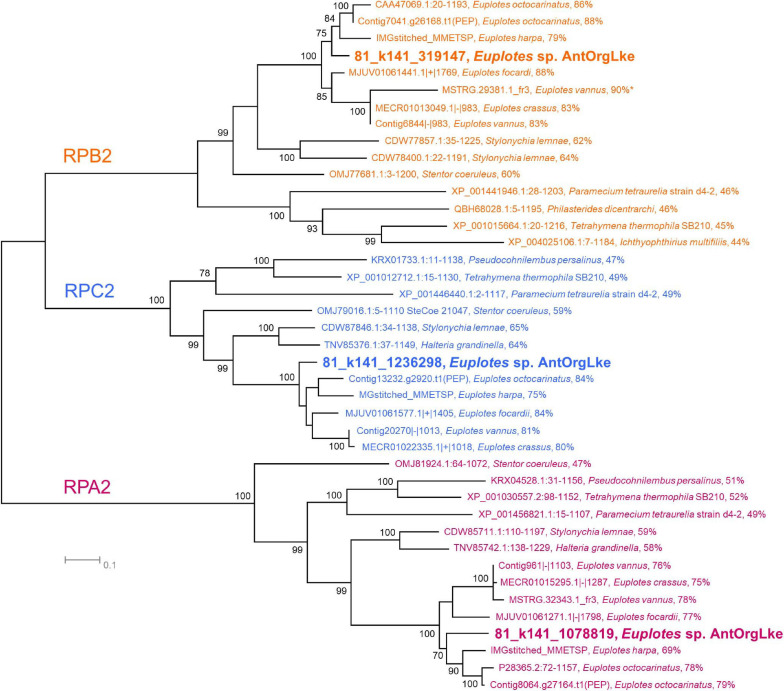
Phylogeny of *Euplotes* sp. AntOrgLke. Unrooted maximum likelihood phylogeny of RNA polymerase subunit II proteins from members of Ciliophora showing *Euplotes* sp. AntOrgLke clustering with members of the *Euplotes* genus. Within the cluster for each RNA polymerase type (RPB, RPC, and RPA), the percent identity between *Euplotes* sp. AntOrgLke protein and an individual protein is shown after the species name. Bootstrap values ≥ 70 are shown next to individual nodes, and protein sequences are available in Supplementary Dataset 5. A total of 41 RNA polymerase subunit II amino acid sequences were used in analysis. Positions with less than 80% site coverage were eliminated, and 944 positions remained in the final dataset, with the exception of MSTRG.29381.1_fr3, *Euplotes vannus* which was a partial sequence (283 aa) and is marked with an*.

*Euplotes* sp. AntOrgLke had 97% 18S rRNA identity to *Euplotes* cf. *antarcticus* and *E. vanleeuwenhoeki*. Tree topology was consistent for all three RNA polymerase sequences (Figure 3) and 18S rRNA sequence (Supplementary Figure 3), and *Euplotes* sp. AntOrgLke seems to be a member of *Euplotes* Clade A (Syberg-Olsen et al., 2016; Boscaro et al., 2018; Serra et al., 2020). The AAI calculated from available *Euplotes* genomic data (six species, including *Euplotes* sp. AntOrgLke) ranged from 49 to 91%, with *Euplotes* sp. AntOrgLke sharing 53–57% with the other five species (Supplementary Table 8). Thus, our data indicate *Euplotes* sp. AntOrgLke is likely a novel Antarctic member of the genus *Euplotes*, and *Ca.* Organicella is a verrucomicrobial endosymbiont of a ciliate species known as *Ca.* Pinguicoccus (Serra et al., 2020). *E. vanleeuwenhoeki*, the host of *Ca.* Pinguicoccus, is a freshwater ciliate (Serra et al., 2020), whereas Organic Lake is hypersaline (Franzmann et al., 1987; Yau et al., 2013).

*Euplotes* is a speciose genus of motile, unicellular ciliate found in many aquatic environments (Boscaro et al., 2019), including Organic Lake, where it was previously detected based on SSU rRNA sequences (Yau et al., 2013). *Euplotes* species have a propensity to harbor one or multiple endosymbiotic bacteria, with at least six genera and 21 species known to date, all of which reside in the cytoplasm (Boscaro et al., 2019; Serra et al., 2020). The majority of reported *Euplotes* endosymbiont species belong to Proteobacteria and are predominantly members of Burkholderiaceae (e.g., *Polynucleobacter*) and the specialized intracellular clades Rickettsiales and Holosporales (Boscaro et al., 2019). The exception is *Ca.* Pinguicoccus, a member of Verrucomicrobia, and the sole known endosymbiont of *E. vanleeuwenhoeki* (Serra et al., 2020). In *E. vanleeuwenhoeki*, *Ca.* Pinguicoccus cells are located free in the cytoplasm and were frequently observed to be in contact with mitochondria and lipid droplets (Serra et al., 2020). The exact benefit of *Ca.* Pinguicoccus to its ciliate host is unclear, although it is unlikely to be nutritional (see *Ca. Organicella–Euplotes Interactions*, later) (Serra et al., 2020). Similarly, the foundations of the symbiotic relationship between proteobacterial endosymbionts and *Euplotes* are unclear, including those that are essential symbionts (*Polynucleobacter*, *Ca.* Protistobacter, and *Ca.* Devosia) and accessory symbionts, with the latter possibly being parasitic (Boscaro et al., 2013, 2019).

### *Ca.* Organicella–*Euplotes* Interactions

**FIGURE 4:**
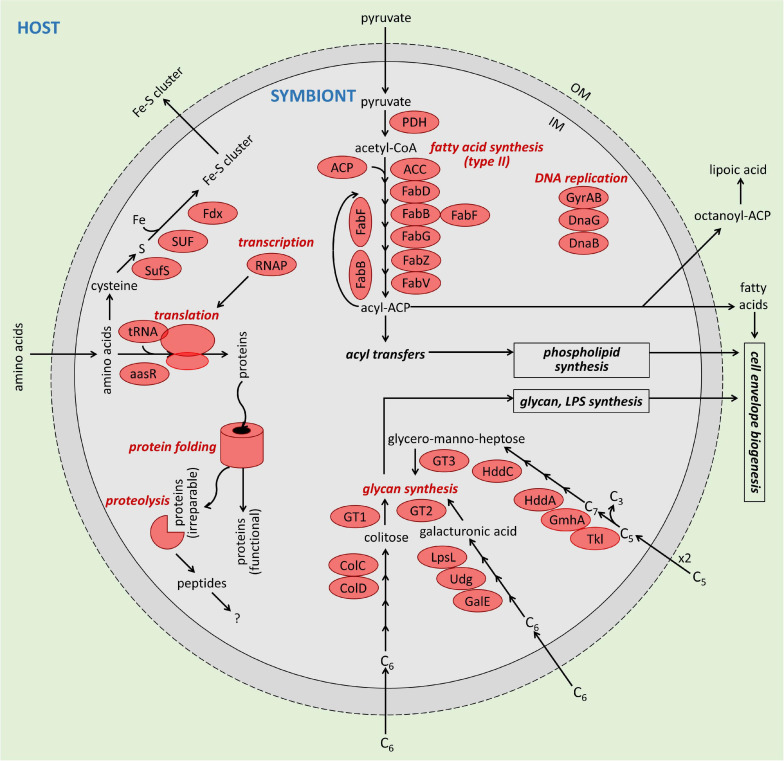
Depiction of function of *Candidatus* Organicella extenuata within *Euplotes* sp. AntOrgLke. The potential metabolic capacities of *Ca.* Organicella extenuata are limited to pyruvate oxidation; type II fatty acid biosynthesis; iron-sulfur (Fe-S) cluster assembly; and conversion, activation, and transfer of hexose and heptose sugars. The *Ca.* Organicella extenuata MAG lacks any identifiable genes for glycolysis, tricarboxylic acid cycle (aside from pyruvate oxidation), pentose phosphate pathway, respiration, fermentation, ATP generation (either by oxidative phosphorylation and ATP synthase, or substrate-level phosphorylation), or synthesis of phospholipids (aside from fatty acids), amino acids, nucleic acids, or vitamins. There were no identifiable transporter genes. Processes, pathways, and enzymes that were inferred to be functional in *Ca.* Organicella extenuata are shaded in red. OM, outer membrane; IM, inner membrane; LPS, lipopolysaccharide. Fatty acid synthesis (type II): PDH, pyruvate dehydrogenase; CoA, coenzyme A; ACP, acyl carrier protein; ACC, acetyl-CoA carboxylase complex; FabD, malonyl-CoA-ACP-transacylase; FabB and FabF, 3-oxoacyl-ACP synthase; FabG, 3-oxoacyl-ACP reductase; FabZ, 3-hydroxyacyl-ACP dehydratase; FabV, enoyl-ACP reductase. Glycan synthesis: GT, glycosyltransferase (three different GT); ColD, GDP-4-keto-6-deoxy-D-mannose 3-dehydratase; ColC, GDP-L-colitose synthase; Udg, UDP-glucose 6-dehydrogenase; LpsL, UDP-glucuronate epimerase; Tkl, transketolase; GmhA, phosphoheptose isomerase; HddA, D-glycero-α-D-manno-heptose 7-phosphate kinase; HddC, D-glycero-α-D-manno-heptose 1-phosphate guanylyltransferase. DNA replication: GyrAB, DNA gyrase; DnaG, DNA primase; DnaB, replicative DNA helicase. Transcription: RNAP, RNA polymerase. Translation: tRNA, transfer RNA; aaRS, aminoacyl tRNA synthetases. Fe-S cluster assembly: Fdx, ferredoxin; SufS, cysteine desulfurase; SUF, Fe-S cluster assembly complex (SufCBD, SufU, SufT). In this reconstruction, pyruvate is supplied by the host and the sole purpose of PDH is to provide the acetyl-CoA precursor for fatty acid synthesis. The fatty acid synthesis pathway is functionally complete in *Ca.* Organicella extenuata, with FabF or FabB substituting for missing FabH. There are three pathways involved in synthesis of heptose (glycero-manno-heptose) or hexose (galacturonic acid; colitose) subunits of lipopolysaccharide glycans in *Ca.* Organicella extenuata, but none of these pathways are complete, and all depend on exogenous precursors.

One possibility is that *Ca.* Organicella provides Fe-S clusters and fatty acids to its host as the foundation for a mutualistic symbiosis (Figure 4). This is pertinent to *Euplotes*, in which, as in other ciliates, the mitochondrial genome does not encode these functions. We identified 41.8 kb of *Euplotes* sp. AntOrgLke mitochondrial genome sequence—a comparable length to the mitochondrial genome sequences reported for other *Euplotes* species (de Graaf et al., 2009; Serra et al., 2020). Like the mitochondrial genomes of *E. crassus, E. minuta*, and *E. vanleeuwenhoeki*, that of *Euplotes* sp. AntOrgLke has genes that encode electron transport chain proteins, ribosomal proteins, rRNA, tRNA, and a cytochrome *c* assembly protein, along with multiple genes that have no known function, but no identifiable Fe-S cluster or fatty acid synthesis genes (Supplementary Table 7; Pritchard et al., 1990; de Graaf et al., 2009; Swart et al., 2011; Johri et al., 2019; Serra et al., 2020). Within the genus *Euplotes*, the mitochondrial genetic code includes a single stop codon (UAA), a single unused codon (UAG), and tryptophan-encoding UGA (Pritchard et al., 1990; Burger et al., 2000; Brunk et al., 2003; de Graaf et al., 2009; Swart et al., 2011). By comparison, in *Ca.* Organicella, UGA is reassigned to Trp, whereas both UAA and UAG are stop codons.

The *Ca.* Organicella MAG encodes ferredoxin and sulfur utilization factor (SUF) proteins involved in Fe-S cluster biogenesis (SufCBD, SufU, and SufT), including cysteine desulfurase (SufS) for the mobilization of sulfur from cysteine (Selbach et al., 2014; Supplementary Table 3). In eukaryotes, the iron–sulfur cluster (ISC) and SUF pathways are the dominant Fe-S cluster synthesis pathways, with ISC assembly proteins located in the mitochondria, whereas SUF assembly proteins are localized to plastid organelles (Kispal et al., 1999; Tsaousis, 2019), the latter including chloroplasts and apicoplasts (Takahashi et al., 1986; Lill and Mühlenhoff, 2005; Lim and McFadden, 2010; Gisselberg et al., 2013), although, in certain protists, SUF assembly proteins are located in the cytoplasm (Tsaousis et al., 2012; Karnkowska et al., 2016). Typical of eukaryotes, *Euplotes* sp. AntOrgLke encodes homologs of ISC proteins inferred to be present in the model ciliate *Tetrahymena thermophila*, including cysteine desulfurase (Nfs1), ferredoxin (Yah1), and ferredoxin reductase (Arh1) (Supplementary Table 9); ISC assembly would occur in the mitochondrion and depend on nuclear-encoded enzymes (Smith et al., 2007). The SUF system of *Ca.* Organicella could therefore function as a complementary Fe-S cluster assembly system to ISC. The SUF system is more resistant to reactive oxygen species than the ISC system (Santos-Garcia et al., 2014); thus, the SUF system encoded by *Ca.* Organicella may be especially important to the host under oxidative stress conditions in response to the degradation of Fe-S clusters of host proteins (Tsaousis, 2019). The SUF system may be especially relevant to *Euplotes* sp. AntOrgLke in Organic Lake and the other Vestfold Hills lakes due to the prevailing environmental conditions (high oxygen concentrations; freezing temperatures; enhanced UV irradiation; Supplementary Figure 1) that promote the production of reactive oxygen species (Ricci et al., 2017).

*Ca.* Organicella also encodes an almost complete suite of genes for bacterial type II fatty acid synthesis (FASII), except for FabH, an enzyme involved in fatty acid elongation (see *Supplementary Text – Pyruvate Oxidation and Fatty Acid Synthesis*). It is likely that another condensing enzyme involved in fatty acid elongation encoded in *Ca.* Organicella (FabB or FabF) would substitute for FabH, as inferred for *Ca.* Wigglesworthia, which similarly lacks FabH but otherwise encodes a complete FASII pathway (Zientz et al., 2004; Parsons and Rock, 2013). In support of this, *Escherichia coli* and *Lactococcus lactis* mutants that lack *fabH* are still capable of synthesizing fatty acids (Morgan-Kiss and Cronan, 2008; Yao et al., 2012). For *Ca.* Organicella, the acetyl-CoA precursor for straight-chain fatty acid biosynthesis would be generated using a pyruvate dehydrogenase (PDH) complex, presumably using pyruvate acquired from the host (Figure 4).

Many protists depend on the fatty acids provided by mitochondrial FASII for processes such as lipoylation of essential enzymes or for incorporation into phospholipids; despite having their own cytoplasmic FAS apparatus (FAS type I), these eukaryotes depend on the fatty acids provided by mitochondria (Stephens et al., 2007; Hiltunen et al., 2009). However, as in other ciliates, the mitochondrial genome of *Euplotes* lacks genes associated with FASII (Pritchard et al., 1990; Burger et al., 2000; Brunk et al., 2003; Swart et al., 2011; Johri et al., 2019). Thus, we propose the hypothesis that *Ca.* Organicella provides fatty acids to the host for these essential purposes.

Another possibility is that fatty acids are supplied to the host in a nutritional capacity. For example, there is evidence that fatty acids synthesized by *Ca.* Blochmannia floridanus form part of the symbiont’s nutritional support to its host (carpenter ant *Camponotus chromaiodes*) during periods when the insect host is feeding on sugar-rich exudates (Zientz et al., 2004; Fan and Wernegreen, 2013). However, we regard this as unlikely, given that it has been predicted that nutritional symbioses are not likely to be necessary for heterotrophic algal and bacterial feeders such as *Euplotes* that can probably obtain all their required nutrients from their diets (Boscaro et al., 2013, 2019; Serra et al., 2020).

It is also possible that FASII in *Ca.* Organicella contributes to its own cellular requirements, including lipoylation of its own PDH and providing precursors for modification of its own cell envelope (Figure 4). In addition to encoding a functionally complete FASII pathway, 17 *Ca.* Organicella genes are predicted to be involved in the biosynthesis of precursors for lipopolysaccharide components: nine proteins are implicated in the biosynthesis of heptose and hexose units (although we could not reconstruct complete pathways), and eight proteins are glycosyltransferases that may be involved in the transfer of nucleotide-activated sugars to construct glycan chains (Supplementary Table 3; *Supplementary Text – Glycan Synthesis*). Obligate endosymbionts with genomes < 500 kbp typically have few if any genes for cell envelope biogenesis, with these pathways being especially prone to loss (McCutcheon and Moran, 2012; Moran and Bennett, 2014; Brown et al., 2015). *Ca.* Organicella lacks acyltransferases necessary for transferring acyl-ACP to glycerol-3-phosphate to produce phosphatidic acid, the phospholipid precursor in bacteria (Yao et al., 2012), and there are no identifiable genes for the biosynthesis of the glycerophosphate backbone or headgroups of phospholipids or for the 3-deoxy-D-manno-octulosonic acid-lipid A (Kdo_2_-lipid A) precursor of lipopolysaccharides (Wang et al., 2015).

Thus, *Ca.* Organicella, as in other endosymbionts with highly reduced genomes, is assumed to rely entirely on host-derived membranes (Baumann, 2005; McCutcheon and Moran, 2012; Husnik and McCutcheon, 2016). The presence of lipopolysaccharide- and other cell-wall-related genes is not unusual for symbiotic bacteria with larger genomes (Zientz et al., 2004; Nikoh et al., 2011); for example, the insect endosymbionts *Ca.* Wigglesworthia and *Ca.* Blochmannia (both between 615 and 706 kbp) encode the majority of genes necessary for the synthesis of a normal gram-negative cell wall, including phospholipids and lipopolysaccharides (Akman et al., 2002; Gil et al., 2003; Zientz et al., 2004). Additionally, certain obligately symbiotic bacteria with larger genomes (>600 kb) retain a complete set of FASII genes (Akman et al., 2002; Gil et al., 2003; Nikoh et al., 2011; Lamelas et al., 2011; Chong and Moran, 2018). In these symbionts, the retention of genes necessary for the synthesis of a normal Gram-negative cell wall (including lipopolysaccharides) is possibly for protection against the host and/or reflects a relatively recent symbiotic association (Akman et al., 2002; Gil et al., 2003). The latter does not apply to *Ca.* Organicella, with the extreme reduction in genome size reflecting an ancient symbiosis (Serra et al., 2020).

Nevertheless, *Ca.* Organicella might contribute glycan components to its own cell envelope (including lipopolysaccharides). One possibility is that modifications of the endosymbiont cell wall confer some protection against the host, such as through variation of fatty acid length or altering the glycan moieties of lipopolysaccharides (core and/or O-specific polysaccharides) using modified sugars by the action of glycosyltransferases (Serra et al., 2020). *Ca.* Pinguicoccus has a very similar genome size and gene composition as *Ca.* Organicella, including retaining homologs of the same glycan/lipopolysaccharide-related genes (see *Comparison of Ca. Organicella and Ca. Pinguicoccus Genomes*, later). *Ca.* Pinguicoccus resides free in the cytoplasm of *E. vanleeuwenhoeki*, and it has been proposed that endosymbionts in the host cytoplasm of eukaryote cells face a less stable and possibly hostile environment compared with those endosymbionts that are enclosed within specialized bacteriocytes or host-derived vesicles (Gil et al., 2003; Wu et al., 2004; Serra et al., 2020). For this reason, *Ca.* Pinguicoccus may exercise some control over the composition of its cell envelope because it is in direct contact with the host cytoplasm (Serra et al., 2020). This might also be true of *Ca.* Organicella, which, based on its close phylogenetic affiliation with *Ca.* Pinguicoccus and having *Euplotes* as the putative host, likely lives in the host cytoplasm.

### Comparison of *Ca.* Organicella and *Ca.* Pinguicoccus Genomes

The genome sizes of *Ca.* Organicella (158,228 bp, 194 genes, 163 CDS) and *Ca.* Pinguicoccus (163,218 bp, 205 genes, 168 CDS; Serra et al., 2020) are similar; note that the protein sequences for the *Ca.* Pinguicoccus NCBI (Accession CP039370) genome sequence were auto-predicted with genetic code 11, but using genetic code 4, we predict a total of 200 genes [five less than reported in Serra et al. (2020)], consisting of 163 CDS, 34 tRNAs, and the 16S, 5S, and 23S rRNA genes (Supplementary Table 1). The two genomes share extensive synteny (Supplementary Figure 4). Although the genomic nucleotide sequences were too divergent to calculate ANI, the AAI between the two symbiont genomes was determined to be 46% (two-way AAI based on 134 proteins, all predicted with genetic code 4). Both genomes retain an almost identical small subset of genes represented across Verrucomicrobia (Serra et al., 2020; Supplementary Table 3). They also share homologous proteins required for DNA replication, transcription, and translation, in common with other endosymbionts, but both lack the catalytic subunit of DNA polymerase (DnaE), which is exceptional among endosymbionts (Serra et al., 2020).

*Ca.* Pinguicoccus encodes the same components of the SUF system and a functionally complete FASII pathway as *Ca.* Organicella, suggesting that *Ca.* Pinguicoccus confers the same benefits to its *Euplotes* host that we infer for *Ca.* Organicella. Of interest is that *Ca.* Pinguicoccus cells were often observed associated with lipid droplets in *E. vanleeuwenhoeki* cytoplasm, raising the possibility of a link between the retention of FASII genes and interaction with the host’s lipids (Serra et al., 2020). *Ca.* Pinguicoccus also encodes homologs of the same glycosyltransferases and heptose- and hexose-related enzymes encoded in *Ca.* Organicella (Supplementary Table 3). Nevertheless, *Ca.* Pinguicoccus retains a putative phospholipid synthesis protein (CDP-diacylglycerol-glycerol-3-phosphate 3-phosphatidyltransferase homolog) not identifiable in *Ca.* Organicella. *Ca.* Pinguicoccus encodes a thioredoxin–thioredoxin reductase system (for maintaining thiol-disulfide redox balance) and NADP-dependent glutamate dehydrogenase (for the reversible oxidative deamination of glutamate), neither of which are identifiable in *Ca.* Organicella. There are also variations between the two genera in the exact complement of ribosomal subunits, aaRS, and initiation factor subunits (Supplementary Table 3), with these components being prone to loss among endosymbionts (Moran and Bennett, 2014). However, *Ca.* Organicella and *Ca.* Pinguicoccus possess the same 34 tRNA genes.

Overall, the data suggest that, as their divergence from a common ancestor had a highly reduced genome, further genomic erosion has occurred independently in *Ca.* Organicella and *Ca.* Pinguicoccus, with differential loss of certain genes, especially those involved in translation. By contrast, SUF, FASII, and certain lipopolysaccharide/glycan-related genes are conserved between the two genera. This suggests that these particular genes play important roles in the interactions of these endosymbionts with their ciliate hosts.

## Conclusion

Many of the smallest bacterial genomes are from insect symbionts that exist as metabolically complementary partnerships within the host (43) (Supplementary Table 1). For example, *Ca.* Nasuia deltocephalinicola (∼112 kbp) and *Ca.* Hodgkinia cicadicola (∼144 kbp) are each co-resident with *Ca.* Sulcia (Bennett and Moran, 2013; McCutcheon et al., 2009), whereas *Ca.* Tremblaya princeps (∼139 kbp) contains *Ca.* Moranella endobia to constitute a nested symbiosis (McCutcheon and von Dohlen, 2011). However, *Ca.* Carsonella ruddii (∼160 kbp) is a lone endosymbiont resident in sap-feeding psyllids (Thao et al., 2000; Nakabachi et al., 2006). Unlike known insect symbionts with highly reduced genomes (Nakabachi and Ishikawa, 1999; Zientz et al., 2004; Nakabachi et al., 2006; Pérez-Brocal et al., 2006; Bennett and Moran, 2013; Brown et al., 2015; Gil et al., 2018), *Ca.* Organicella and *Ca.* Pinguicoccus lack any capacity for the biosynthesis of amino acids or vitamins (Serra et al., 2020). Thus, as with the Ca. Pinguicoccus-*E. vanleeuwenhoeki* symbiosis, there is no reason to assume that *Ca.* Organicella exists as part of a co-symbiotic partnership, especially considering that none of the genes encode enzymes involved in amino acid or vitamin biosynthesis, as is typical for such consortia (McCutcheon et al., 2009; McCutcheon and von Dohlen, 2011). The absence of a nutritional basis of a proposed *Ca.* Organicella*-Euplotes* symbiosis likely reflects the algivorous and bacterivorous nature of the ciliate host (Serra et al., 2020), in contrast to insects with specialized and nutritionally unbalanced diets. Instead, we propose that *Ca.* Organicella and *Ca.* Pinguicoccus provide SUF Fe-S clusters and FASII fatty acids as essential molecules to the host, with FASII replacing a lost mitochondrial function in *Euplotes*. Additionally, the ciliate host would possess dual Fe-S cluster biogenesis systems, with the SUF system provided by endosymbiotic Verrucomicrobia.

*Ca.* Organicella was identified as possibly being an endosymbiont by virtue of having unusual coding parameters (Ivanova et al., 2014) rather than by searching our metagenome data for symbionts. Previous metagenomic screening of multiple *Euplotes* strains and their resident bacteria did not recover symbionts that belong to phylum Verrucomicrobia (Boscaro et al., 2019). In the study, the identification of putative symbionts in *Euplotes*-based metagenome data was based on bacterial taxa that were referrable to known clades of exclusively intracellular bacteria (e.g., Rickettsiales) or related to previously described protist symbionts (e.g., *Polynucleobacter*) (Boscaro et al., 2019); thus, any verrucomicrobial symbionts might have been overlooked, especially if they were present at low coverage. Targeted hosts and/or symbiont reference genomes have been used extensively for identifying both ecto- and endo-symbionts of a broad range of taxa, including magnetotactic bacteria of marine protists (Monteil et al., 2019), gut symbionts of hadal snailfish (Lian et al., 2020) and phytophagous stink bugs (Kashkouli et al., 2020), and symbionts of pea aphids (Guyomar et al., 2018) and scleractinian corals (Shinzato et al., 2014). The discovery of *Ca.* Pinguicoccus arose through the development of a “next-generation taxonomy” approach for assessing symbiont–host associations that combines “bio-taxonomy tools, classical morphology, ultrastructure, molecular phylogeny, genomics, and bioinformatics” (Serra et al., 2020). The study focused on *Euplotes* as a model protist “holobiont,” in the process of identifying *Ca.* Pinguicoccus. Being a host-based approach to endosymbiont discovery, the “next-generation taxonomy” approach is applicable to known symbiotic communities. Software (MinYS) has also recently been reported to specifically identify symbionts from genome assemblies of symbiotic communities by using reference genomes (Guyomar et al., 2020). Although genetic code 4 (UGA stop codons recoded to tryptophan) has been reported to only occur rarely (Yamao et al., 1985; Knight et al., 2001; McCutcheon et al., 2009), our findings raise the enticing prospect that searching contigs and MAGs for this recoding may reveal new symbionts, including members of the verrucomicrobial “Nucleococcus cluster” (Figure 1).

## Data Availability Statement

The datasets presented in this study can be found in online repositories. The repository and accession numbers are: NCBI (*Euplotes* sp. AntOrgLke MAG, accession: JAGXKF000000000 and PRJNA720161); IMG (https://img.jgi.doe.gov/) (*Ca.* Organicella extenuata MAG: Scaffold Ga0307966_1000010; *Euplotes* sp. AntOrgLke mitochondrial genome: 2 Scaffolds, Ga0307966_1001133 and Ga0307966_1001206).

## Ethics Statement

Written informed consent was obtained from the individual(s) for the publication of any potentially identifiable images or data included in this article.

## Author Contributions

TW, MA, NI, MH, and RC conceived the study, analyzed the data, and conducted data interpretation. SH performed Sterivex filter DNA extractions. AH and SB spent 18 months in Antarctica running the 2013–2015 expedition. TW, MA, and RC wrote the manuscript with input from all other co-authors. All authors have read and approved the manuscript submission.

## Conflict of Interest

The authors declare that the research was conducted in the absence of any commercial or financial relationships that could be construed as a potential conflict of interest.

## Footnotes

1 https://github.com/JCSzamosi/SparCC3

2 https://blast.ncbi.nlm.nih.gov/

3 https://www.ncbi.nlm.nih.gov/Taxonomy/Utils/wprintgc.cgi#SG4
