# Supplementary material for: Genome Analysis of a Verrucomicrobial Endosymbiont With a Tiny Genome Discovered in an Antarctic Lake
Source: Front Microbiol. 2021 Jun 1;12:674758. doi: 10.3389/fmicb.2021.674758 (PMC8204192; doi:10.3389/fmicb.2021.674758)
Supplement: Supplementary file 7 [file Data_Sheet_1.pdf]

Supplementary Material

A new type of bacterial endosymbiont with a tiny genome discovered in Antarctica

Timothy J. Williams, Michelle A. Allen, Natalia Ivanova, Marcel Huntemann, Sabrina Haque, Alyce M. Hancock, Sarah Brazendale, and Ricardo Cavicchioli

1 Supplementary Text

tRNA modification

Pyruvate oxidation and fatty acid synthesis

Glycan synthesis

2 Supplementary Figures

Figure S1|Expedition sampling of Antarctic, Vestfold Hills lakes that harbored *Candidatus* Organicella extenuata and *Euplotes* sp. AntOrgLke.

Figure S2|Phylogeny of *Candidatus* Organicella extenuata using concatenated marker genes.

Figure S3|Phylogeny of *Euplotes* sp. AntOrgLke using 18S rRNA gene sequences.

Figure S4|Synteny of *Ca*. Organicella and *Ca*. Pinguicoccus genomes.

3 Supplementary Tables

Table S1|Characteristics of bacteria with highly reduced genomes.

Table S2|Functional annotation of all *Ca.* Organicella extenuata and *Ca*. Pinguicoccus supinus genes. (see accompanying excel file, multiple tabs)

Table S3|Overview of functional capacity of *Ca.* Organicella extenuata and *Ca*. Pinguicoccus supinus inferred from their genomic potential.

Table S4|Sequence identity between the *Ca.* Organicella 16S rRNA gene from different metagenomes. (see accompanying excel file)

Table S5|Detection of *Ca.* Organicella extenuata in Antarctic metagenomes. (see accompanying excel file, multiple tabs)

Table S6|Taxonomy predictions for *Euplotes* sp. AntOrgLke bins and bin abundance data. (see accompanying excel file, multiple tabs)

Table S7|Features of the *Euplotes* sp. AntOrgLke mitochondrial genome, and results from blastp against reference *Euplotes* spp. (see accompanying excel file)

Table S8|Amino acid identity between *Euplotes* species.

Table S9|Iron-sulfur cluster proteins identified in *Euplotes* genome data. (see accompanying excel file)

4 Supplementary Datasets

Dataset S1|Euplotes_MAG.fna.txt (zip file)

Dataset S2|Euplotes_MAG_v_Uniclust90.faa.txt (zip file)

Dataset S3|Euplotes_MAG_v_MERCMMETSP.faa.txt (zip file)

Dataset S4|Euplotes sp. AntOrgLke mitochondrial genome.fasta.txt

Dataset S5|Euplotes_RNAPolymeraseSubunit-II_proteins.fasta.txt

5 Supplementary References

Supplementary Text

tRNA Modification

Ca. Organicella extenuata encodes proteins for modification of the anticodon wobble uridine, using the MnmEG complex, which incorporates aminomethyl and carboxymethylaminomethyl (Armengod et al., 2012; Moukadiri et al., 2014), and MnmA, for thiolation of this same uridine (Kambampati and Lauhon, 2003), with sulfur mobilized by cysteine desulfurase (SufS). *Ca.* Organicella extenuata encodes the enzyme (TilS) required for the conversion of cytidine to lysidine of the AUA codon-specific tRNA(Ile) (Soma et al., 2003). *Ca.* Organicella extenuata also encodes a single protein (TsaD) required for the N6-threonylcarbamoylation of tRNA, an essential modification required for translational fidelity by the ribosome (Wan et al., 2013). In bacteria, the catalytic protein TsaD typically functions as part of an enzyme complex (TsaBCDE) (Deutsch et al., 2012), but in bacteria with highly reduced genomes, TsaD can apparently function alone (Wan et al., 2013).

Pyruvate Oxidation and Fatty Acid Synthesis

As described in the main text, in *Ca.* Organicella extenuata a complete pyruvate dehydrogenase (PDH) complex (Zientz et al., 2004) is encoded for the oxidation of pyruvate to acetyl-CoA. Curiously, there is an additional pair of homologs of PDH E1 α and β subunits, within a gene cluster that also contains genes implicated in LPS synthesis (GalE/Agl12, HddC, HddA, ColD), as well as a B_{12}-binding domain-containing radical SAM protein methyltransferase of unknown function.

Pyruvate for PDH would need to be acquired exogenously, presumably from the host. Acetyl-CoA serves as the precursor for straight-chain fatty acid biosynthesis; in the absence of a substrate-level phosphorylation pathway involving acetyl-CoA (Zientz et al., 2004), we infer that the acetyl-CoA serves exclusively for FASII. Acetyl-CoA generated by PDH is used to construct fatty acids, beginning with acetyl-CoA carboxylase (ACC), which performs the first committed step in FASII to generate malonyl-CoA through the carboxylation of acetyl-CoA (Parsons and Rock, 2013). All ACC subunits were encoded in *Ca.* Organicella extenuata: biotin carboxylase (AccC), biotin carboxyl carrier protein (AccB), and carboxyltransferase (AccAD). The transfer of the malonate group from malonyl-CoA to ACP is carried out by malonyl-CoA-ACP-transacylase (FabD). The acidic ACP, which is essential for shuttling nascent fatty acids between FASII enzymes, is encoded. Additionally, ACP synthase, required for post-translational modification of ACP into the active form (McAllister et al., 2006), was not identifiable in the *Ca.* Organicella extenuata genome; presumably this conversion (along with the 4'-phosphopantetheine moiety derived from CoA) is also provided by the host.

For the elongation phase, the short chain 3-oxoacyl-ACP synthase (3-oxoacyl-ACP synthase 3; FabH) that synthesizes the acetoacetyl-ACP precursor could not be identified in the *Ca.* Organicella

extenuata genome; thus, we posit that another condensing enzyme 3-oxoacyl-ACP synthase 1 or 2 (FabB or FabF, respectively) performs the function of FabH (Zientz et al., 2004; Parsons and Rock, 2013). *Ca.* Organicella extenuata encodes the enzymes required for the elongation of the fatty acid chain: 3-oxoacyl-ACP reductase (FabG); 3-hydroxyacyl-ACP dehydratase (FabZ); enoyl-ACP reductase (FabV). The acyl-ACP produced by this elongation module can be used by the condensing enzymes FabB or FabF to initiate another round of elongation. Although the FASII pathway appears to be functional, the ATP, CoA, and reduced cofactors would need to be acquired exogenously.

Glycan Synthesis

Overall, the *Ca.* Organicella extenuata genome encodes enzymes potentially involved in the generation of precursors for LPS components: fatty acids for lipid A, and glycans for the LPS core and O-antigen. Seventeen *Ca.* Organicella extenuata genes are predicted to be involved in the synthesis of glycan chains. These comprise nine genes implicated in the biosynthesis of heptose or hexose units of LPS and eight putative glycosyltransferases (**Supplementary Table S3**). In bacteria, activated sugar units can also be incorporated into the glycan moieties of S-layer glycoproteins, capsular polysaccharides, and glycolipids, in addition to LPS. Complete heptose- and hexose-related pathways could not be reconstructed in *Ca.* Organicella extenuata, and all depend on exogenous sugar-phosphate precursors. Additionally, the *Ca.* Organicella extenuata glycosyltransferases have low sequence identities to known glycosyltransferases (<26%), as is typical of glycosyltransferases (Samuel and Reeves, 2003).

Heptose sugars, especially glycero-manno-heptoses, are found in the cell surface glycoconjugates of many bacteria, including for modification of lipid A of LPS (Kneidinger et al., 2001; Kneidinger et al., 2002) and as a constituent of the LPS oligosaccharide core (Taylor et al., 2008). Glycero-manno-heptoses are also incorporated into S-layer glycoprotein (Kneidinger et al., 2001) and capsular polysaccharide (Karlyshev et al., 2005). Four *Ca.* Organicella extenuata proteins were implicated in glycero-manno-heptose synthesis. Transketolase generates the D-sedoheptulose-7-phosphate precursor, although the pentose substrates would need to be sourced exogenously. Phosphoheptose isomerase (GmhA) then catalyzes the isomerization of D-sedoheptulose 7-phosphate into D-glycero-D-manno-heptose 7-phosphate. Although a complete pathway could not be reconstructed, *Ca.* Organicella extenuata encodes homologs of two enzymes involved in a GDP-activated heptose pathway for glycero-manno-heptose synthesis: D-glycero-α-D-manno-heptose 7-phosphate kinase (HddA); D-glycero-α-D-manno-heptose 1-phosphate guanylyltransferase (HddC) (Kneidinger et al., 2001).

Five proteins encoded in *Ca.* Organicella extenuata appear to have functions in glycan biosynthesis using hexose units, based on homologous enzymes in bacteria, archaea, and eukaryotes. One protein is a nucleotide-sugar epimerase/dehydratase homolog, possibly UDP-glucose 4-epimerase (GalE). This protein is also homologous to Agl12, a dTDP-glucose-4,6-dehydratase involved in N-glycosylation of the surface layer (S-layer) in archaea (Kaminski et al., 2013; Kaminski and Eichler, 2014). Two other proteins are homologs of enzymes that catalyze consecutive steps in the biosynthesis of UDP-galacturonic acid in bacteria: UDP-glucose 6-dehydrogenase (Udg) and UDP-glucuronate epimerase (LpsL) (Kereszt et al., 1998). UDP-galacturonic acid can be a constituent of the O-antigen of LPS (Samuel and Reeves, 2003), capsular polysaccharide (Kereszt et al., 1998), and S-layer glycans (Broach et al., 2012). Two other proteins are homologs of enzymes that catalyze two consecutive steps in the biosynthesis of the sugar colitose, a constituent of LPS O-antigen: GDP-4-keto-6-deoxy-D-mannose 3-dehydratase (ColD) and GDP-L-colitose synthase (ColC) (Cook et al., 2008). The latter is also homologous to GDP-L-fucose synthase from plants and rhizobia.

a

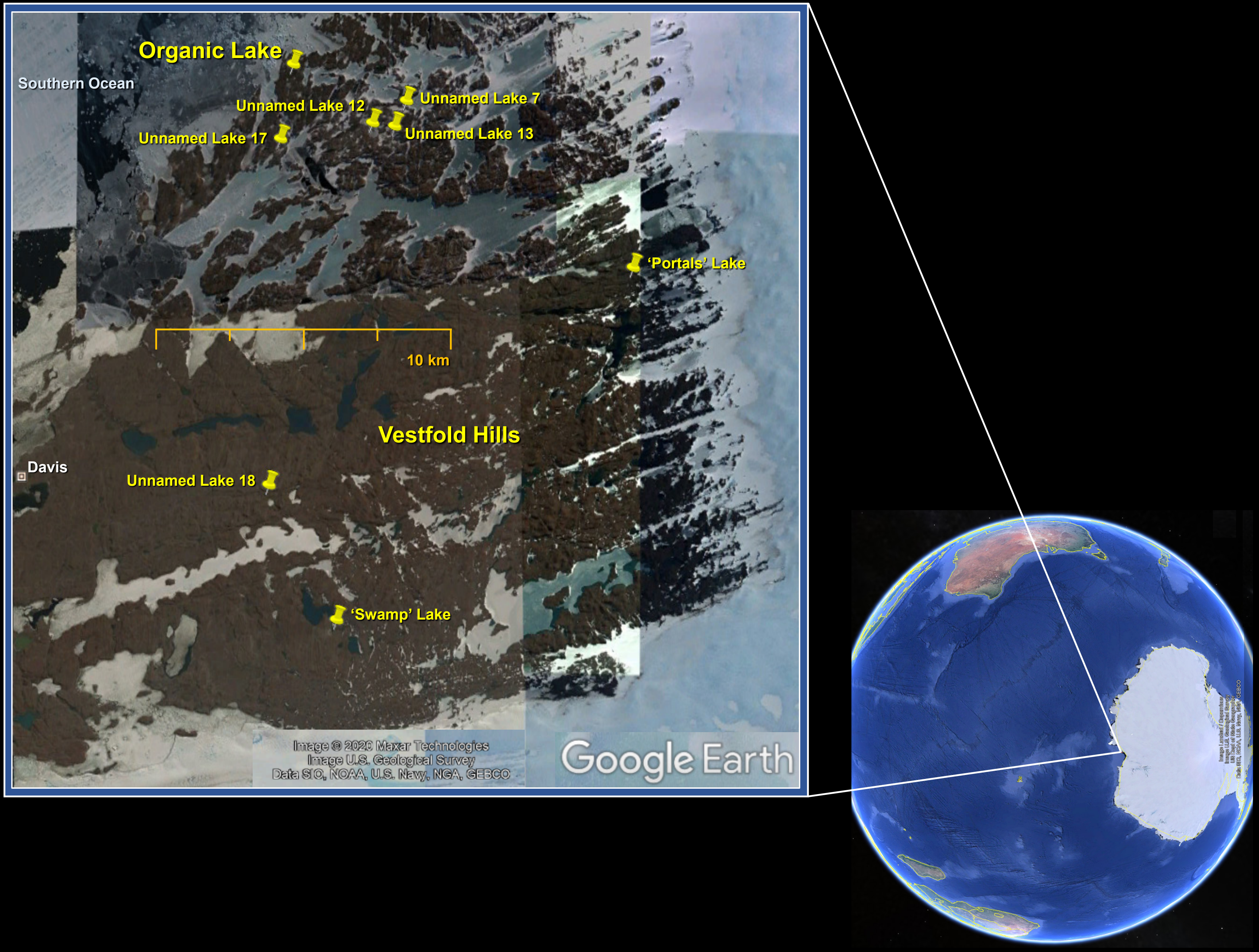

Organic Lake
Southern Ocean
Unnamed Lake 12
Unnamed Lake 7
Unnamed Lake 13
Unnamed Lake 17
'Portals' Lake
10 km
Vestfold Hills
Davis
Unnamed Lake 18
'Swamp' Lake
Image © 2020 Maxar Technologies
Image U.S. Geological Survey
Data SIO, NOAA, U.S. Navy, NGA, GEBCO
Google Earth

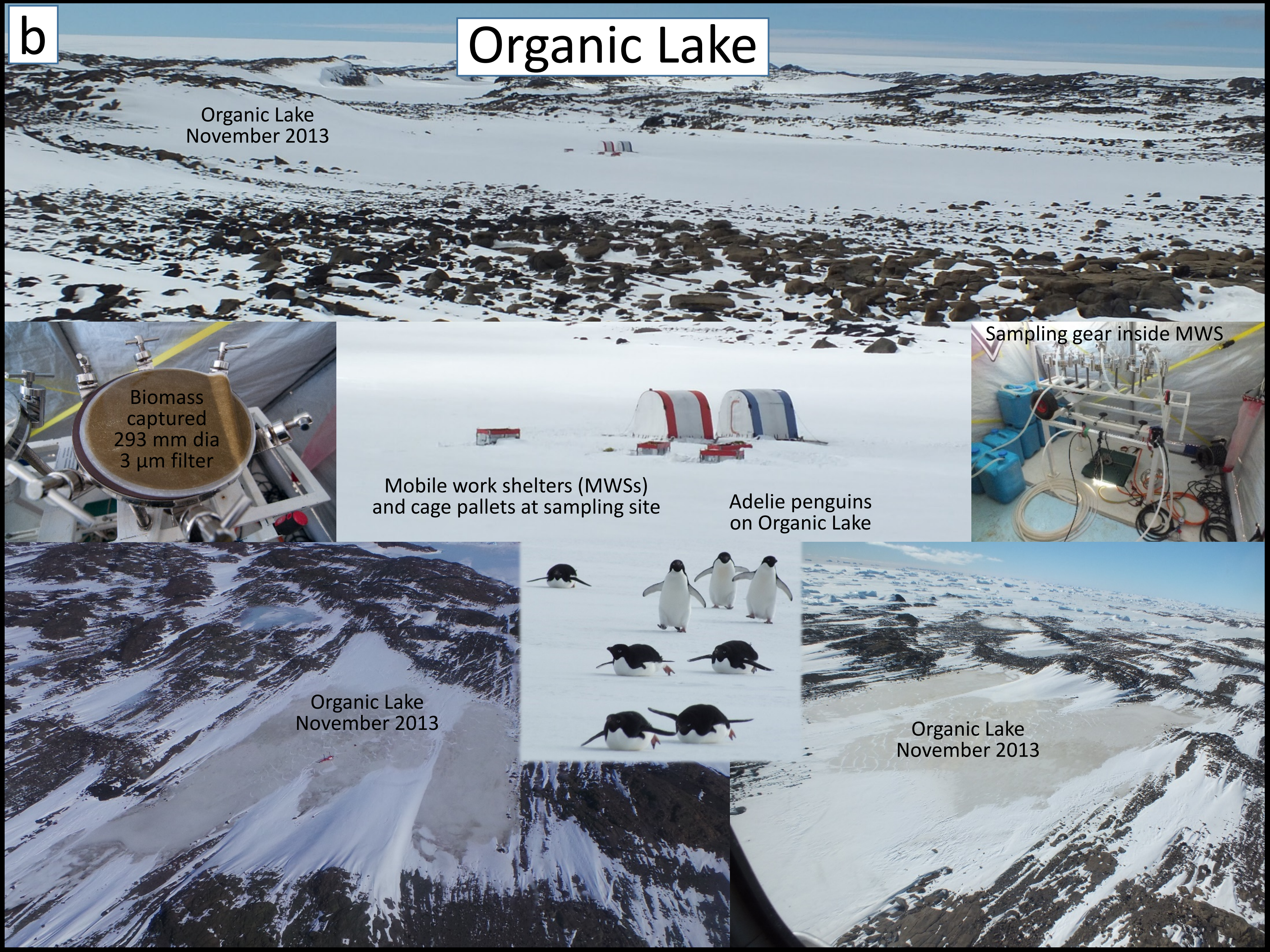
b
Organic Lake
Organic Lake
November 2013
Biomass
captured
293 mm dia
3 µm filter
Mobile work shelters (MWSs)
and cage pallets at sampling site
Adelie penguins
on Organic Lake
Sampling gear inside MWS
Organic Lake
November 2013
Organic Lake
November 2013

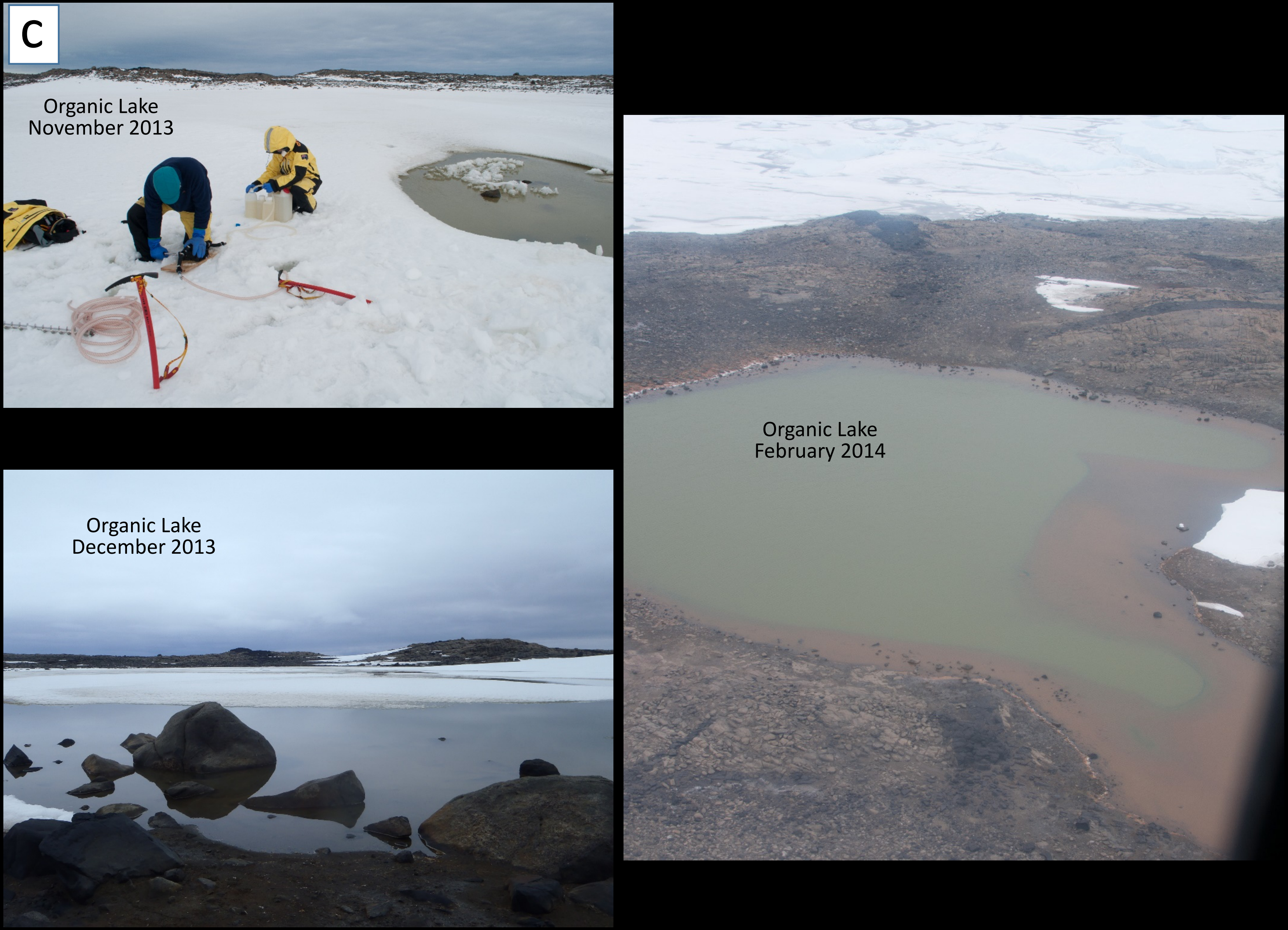
C
Organic Lake
November 2013
Organic Lake
December 2013
Organic Lake
February 2014

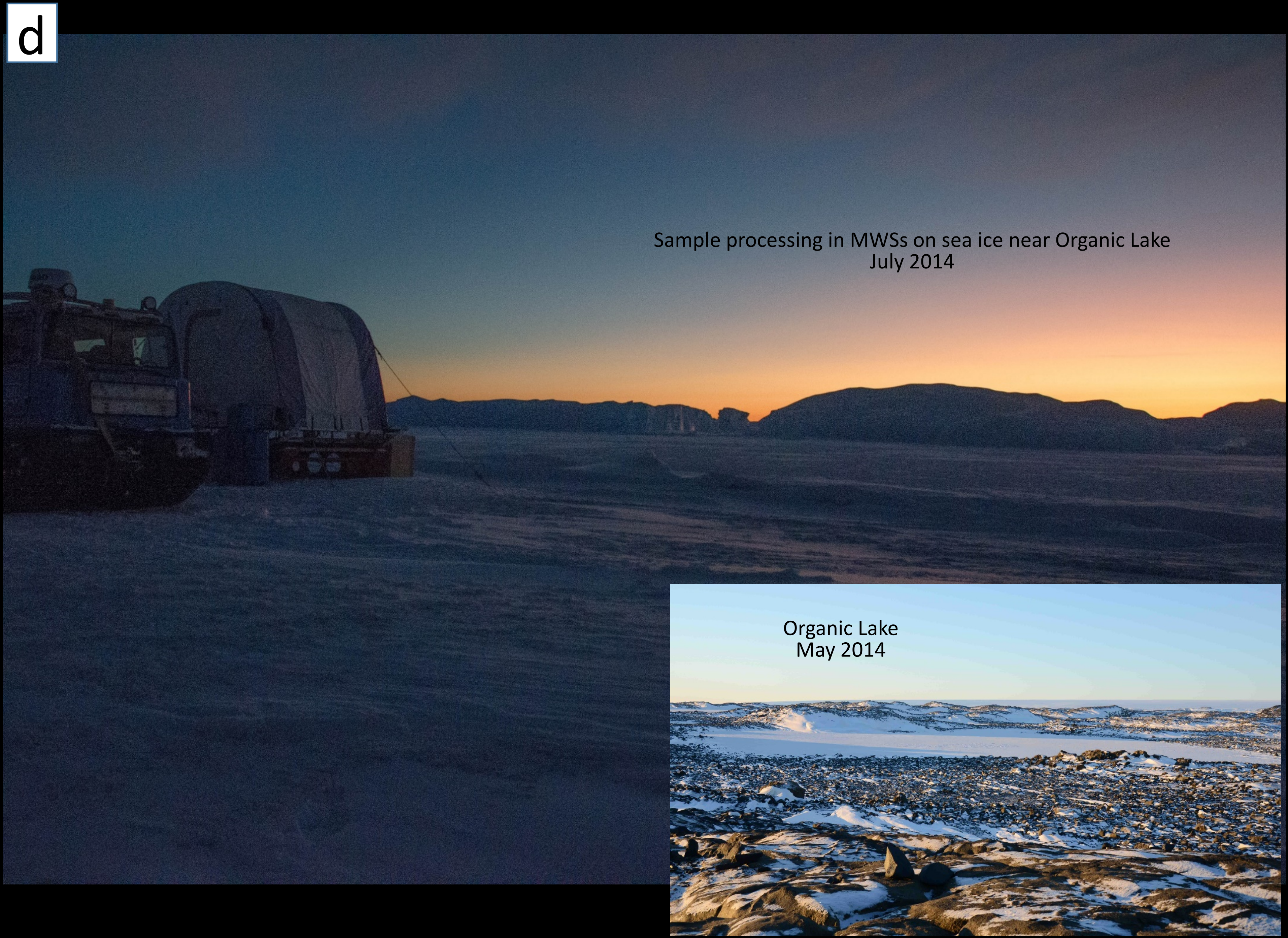
d
Sample processing in MWSs on sea ice near Organic Lake
July 2014
Organic Lake
May 2014

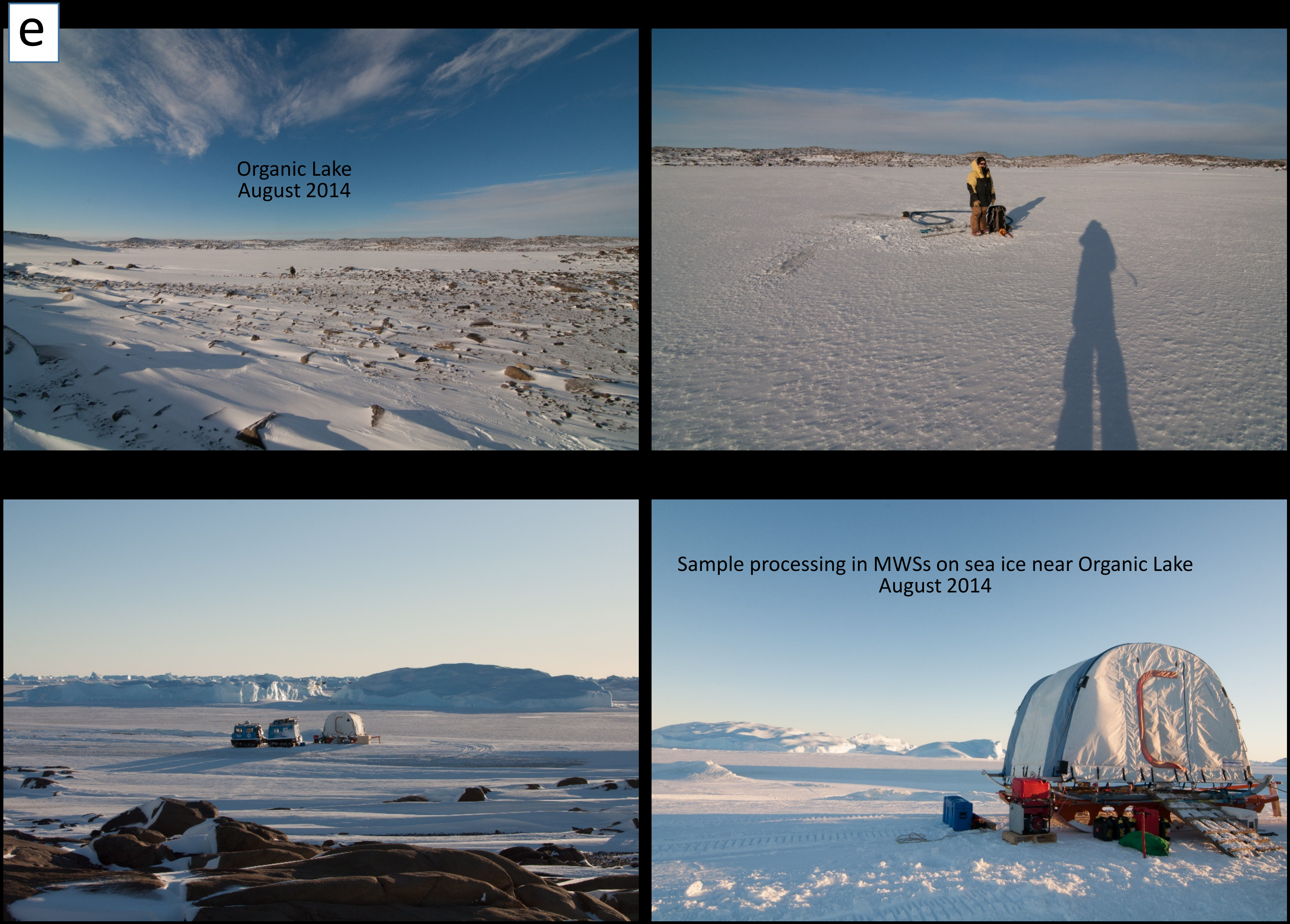
e
Organic Lake
August 2014
Sample processing in MWSs on sea ice near Organic Lake
August 2014

f

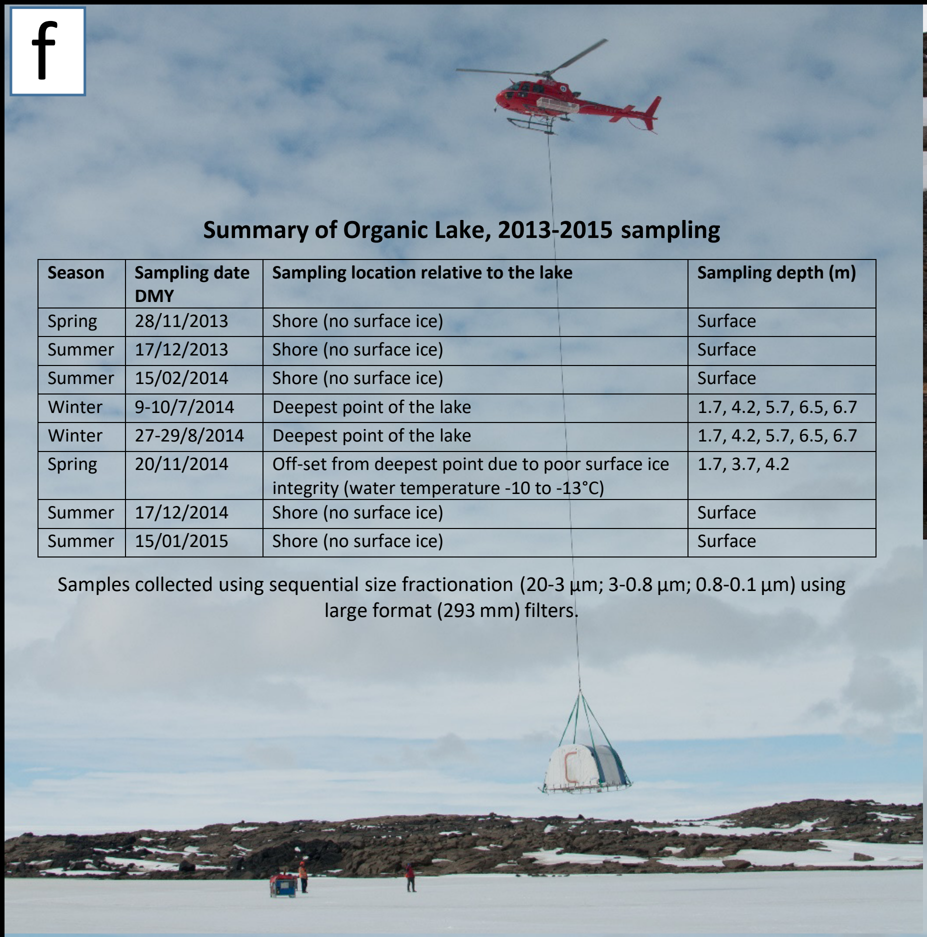

Summary of Organic Lake, 2013-2015 sampling

Season	Sampling date DMY	Sampling location relative to the lake	Sampling depth (m)
Spring	28/11/2013	Shore (no surface ice)	Surface
Summer	17/12/2013	Shore (no surface ice)	Surface
Summer	15/02/2014	Shore (no surface ice)	Surface
Winter	9-10/7/2014	Deepest point of the lake	1.7, 4.2, 5.7, 6.5, 6.7
Winter	27-29/8/2014	Deepest point of the lake	1.7, 4.2, 5.7, 6.5, 6.7
Spring	20/11/2014	Off-set from deepest point due to poor surface ice integrity (water temperature -10 to -13°C)	1.7, 3.7, 4.2
Summer	17/12/2014	Shore (no surface ice)	Surface
Summer	15/01/2015	Shore (no surface ice)	Surface

Samples collected using sequential size fractionation (20-3 µm; 3-0.8 µm; 0.8-0.1 µm) using large format (293 mm) filters.

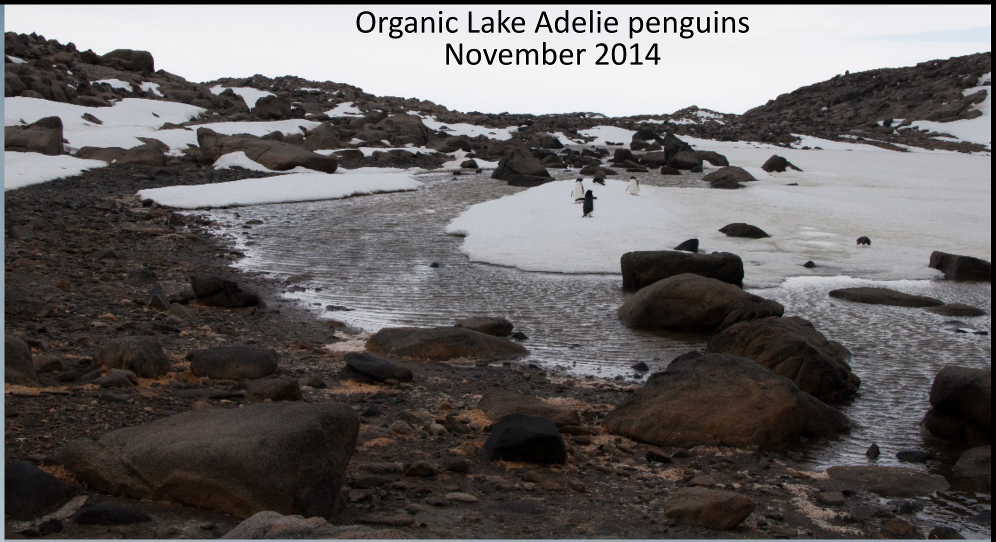

Organic Lake Adelie penguins
November 2014

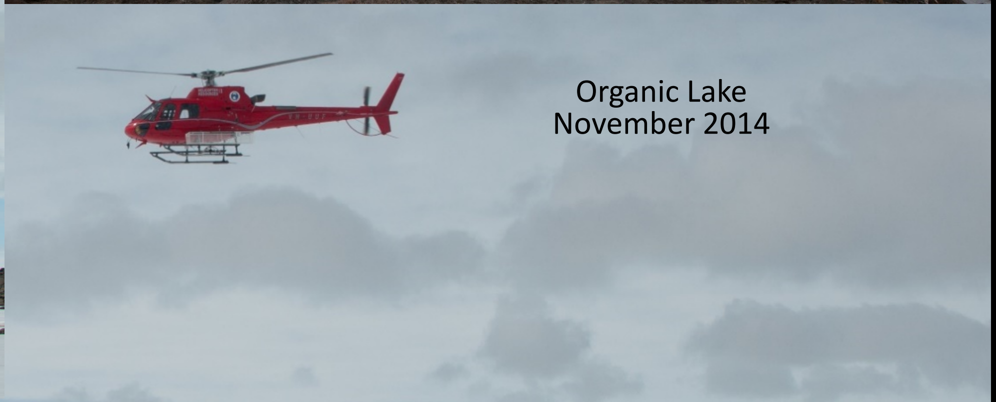

Organic Lake
November 2014

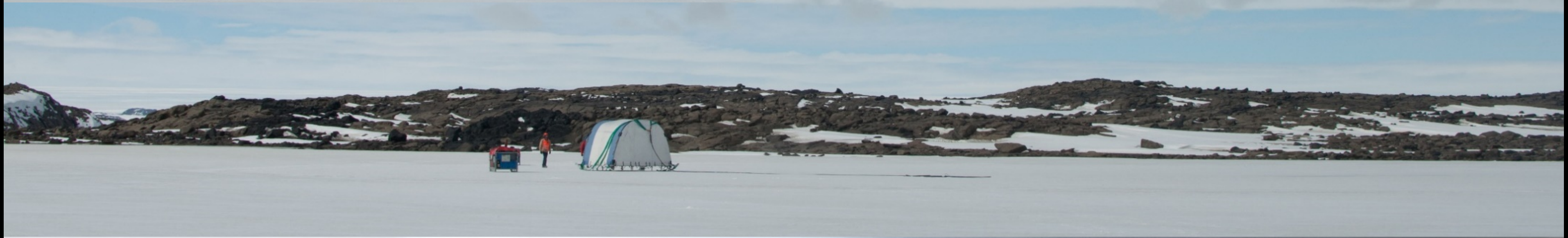

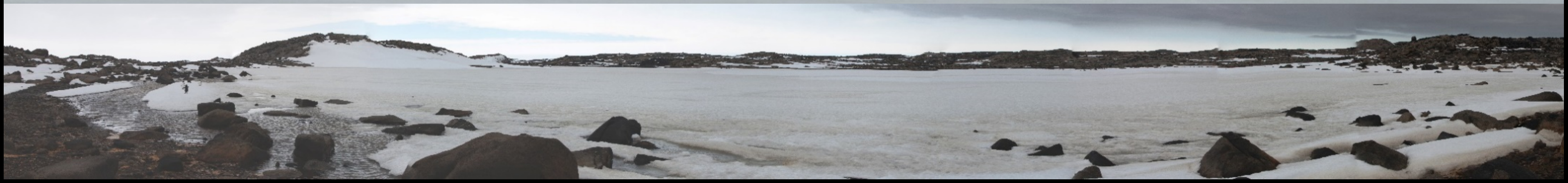

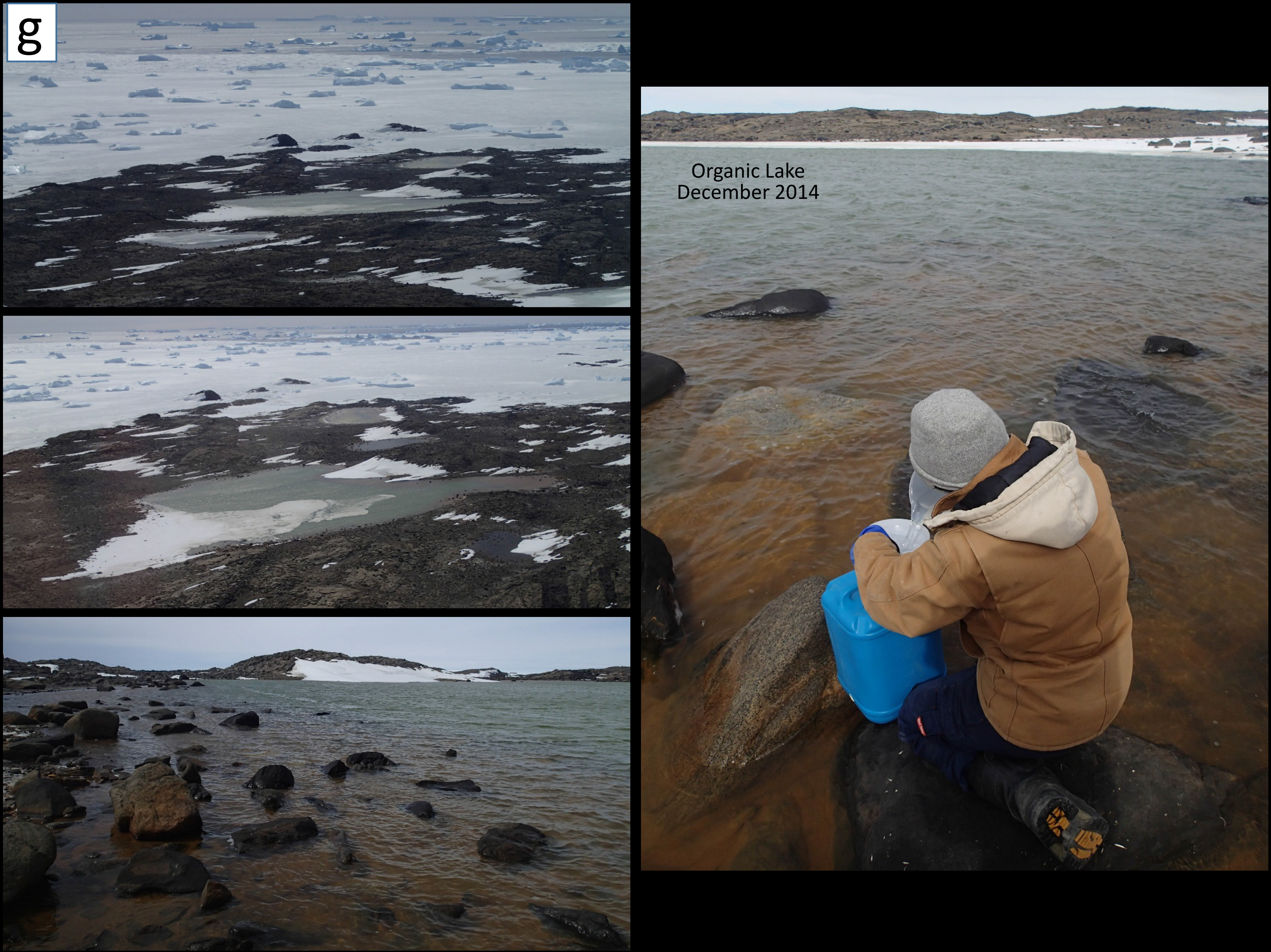
g
Organic Lake
December 2014

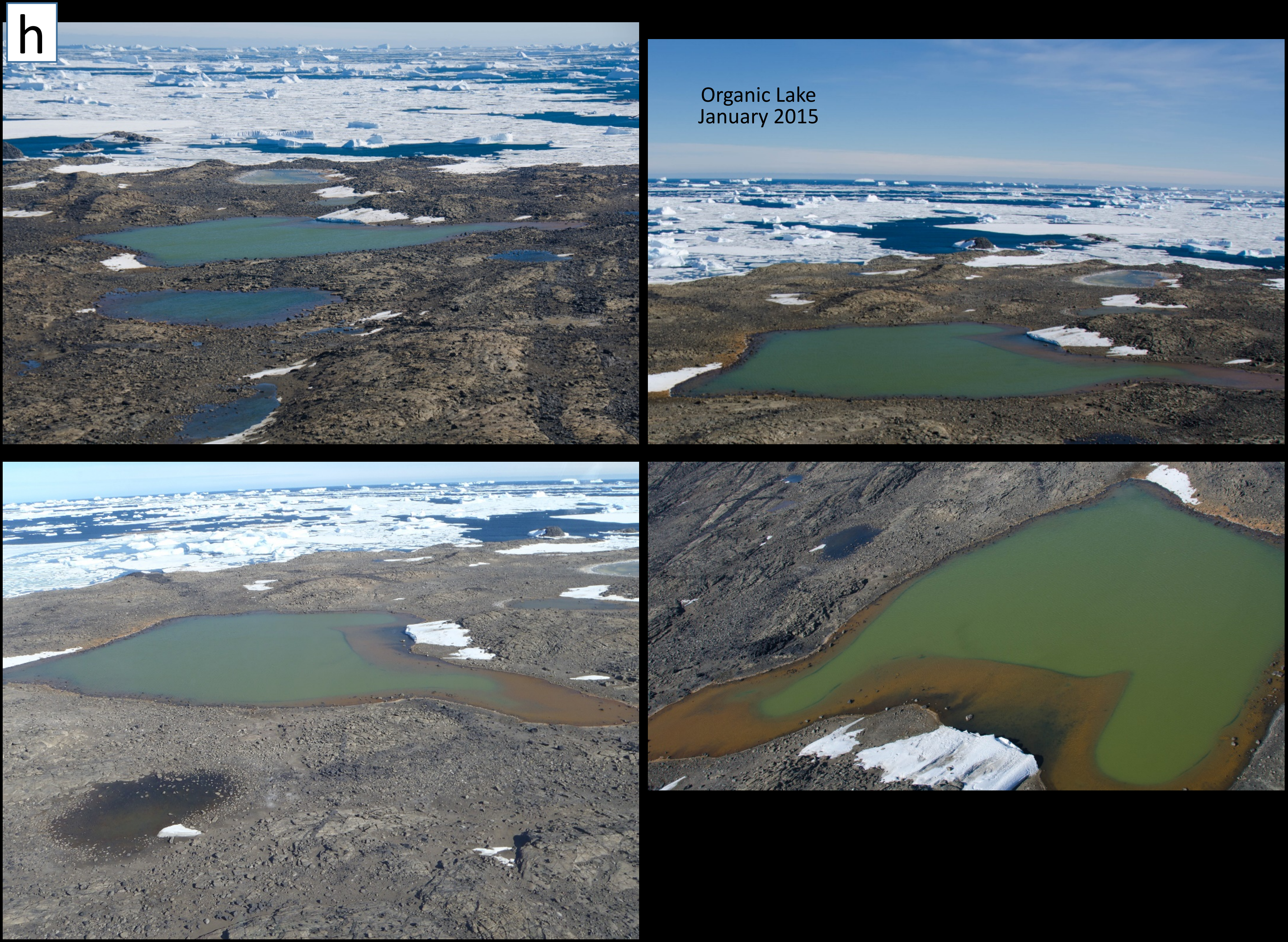
h
Organic Lake
January 2015

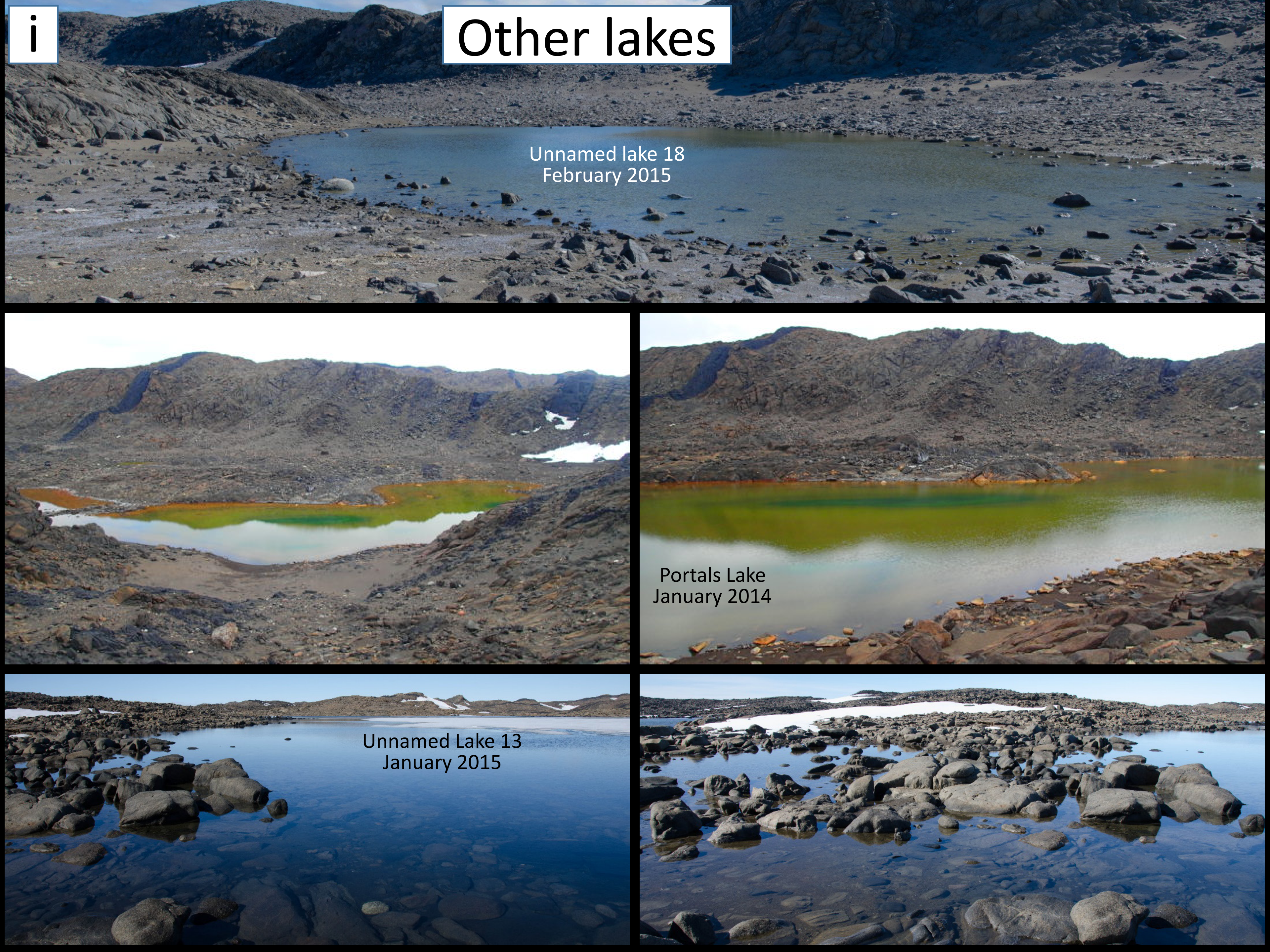
i
Other lakes
Unnamed lake 18
February 2015
Portals Lake
January 2014
Unnamed Lake 13
January 2015

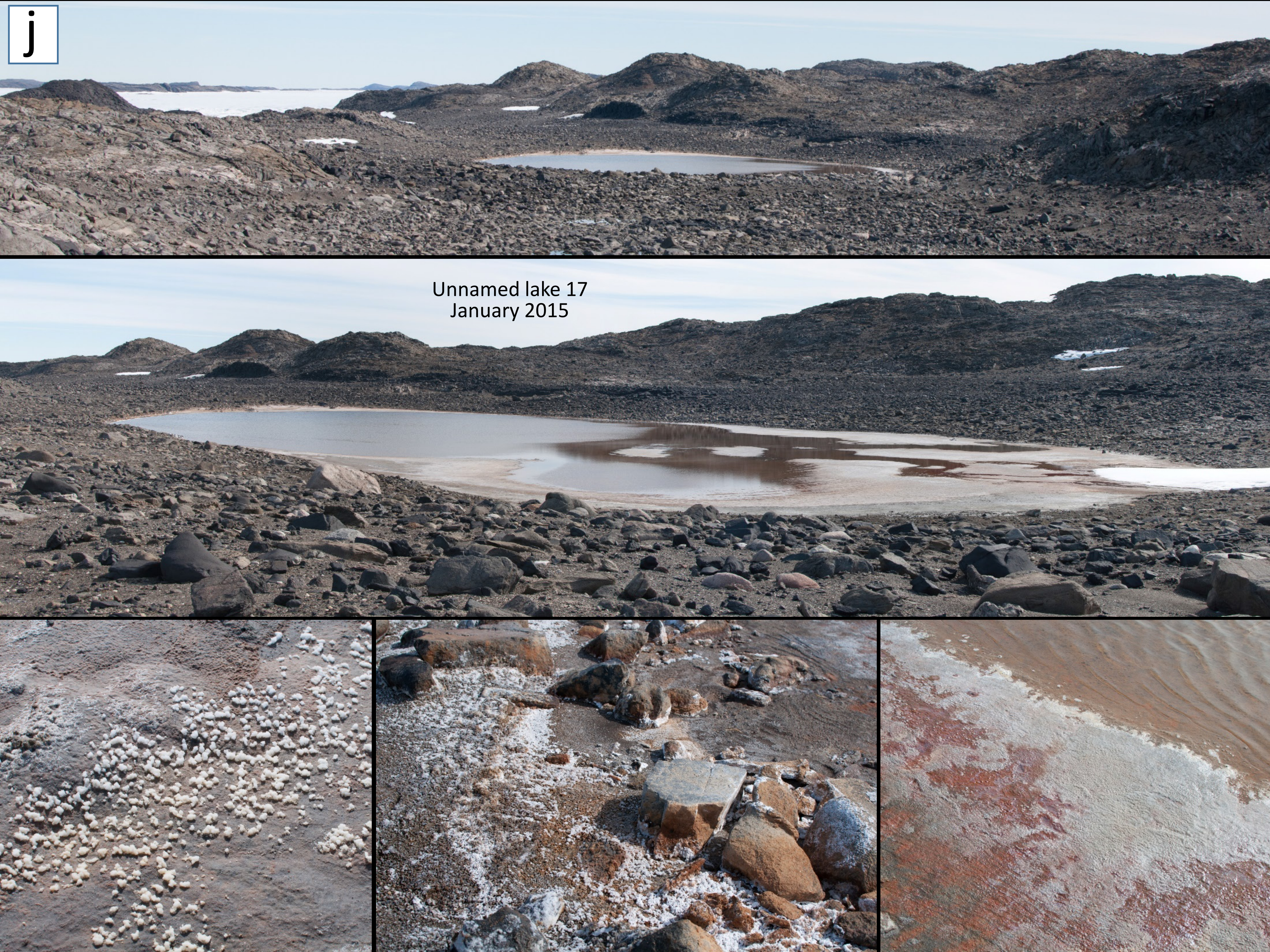
j
Unnamed lake 17
January 2015

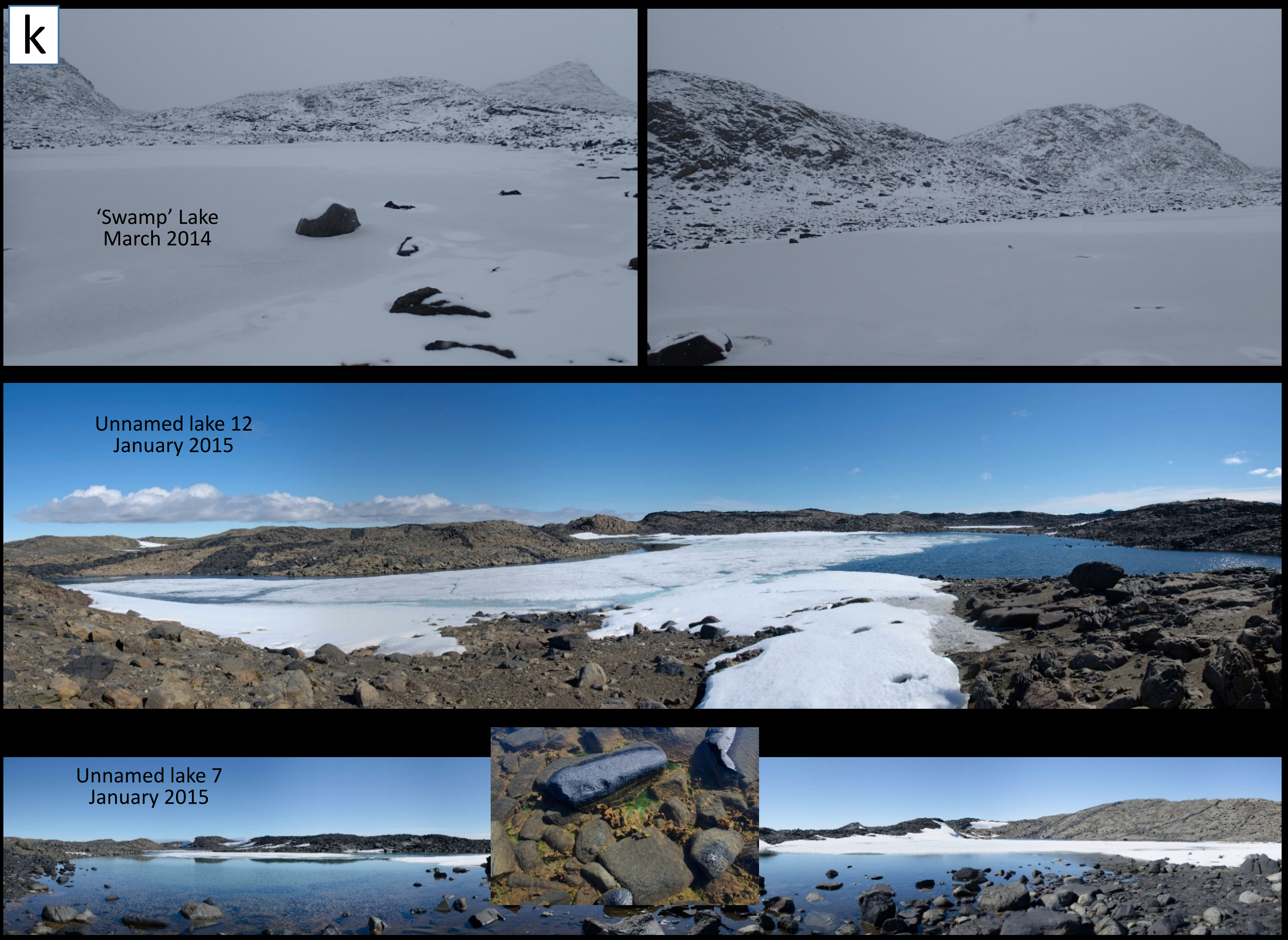
k
'Swamp' Lake
March 2014
Unnamed lake 12
January 2015
Unnamed lake 7
January 2015

I

Metadata for Vestfold Hills lakes, other than Organic Lake, which contained *Organicella* in their metagenomes.

Lake	Location	Sampling date DMY	Water temp. °C	Elevation m	Volume filtered ml *	Density g ml^{-1} / Salinity mS cm^{-1}	Surface area m^2	Lake bottom	Lake shore	Observations
Unnamed Lake 18	S68˚34.923’, E078˚10.200’	1/02/2015	NA	9	1200	1.0059 / 18		Rock, silt	Rock, sand	No ice cover or snow around edges.
‘Portals’ Lake	S68˚31.010’, E078˚28.070’	30/01/2014	5	9	600	1.0325 / 59	12,045	Sand, pebbles	Sand, rock	Shallow with quite yellow/green colouring except in a couple of patches of blue.
Unnamed Lake 13	S68˚28.423’, E078˚15.276’	6/01/2015	7	15	2000	1.0189 / 44		Sand, rock	Sand, rock	Unnamed Lake 13 joined to Unnamed Lake 14. Some Ice on lake 13 and no ice on 14. Sample collected at the point the two lakes join. Very salty – salt crystals growing in puddles of water on rocks surrounding the lakes.
Unnamed Lake 17	S68˚28.711’, E078˚10.77’	7/01/2015	17	7	250	1.0878 / 121		Sand, rock, silt	Sand, rock, silt	No ice on the lake or snow around the edges of the lake. Lake orange/brown in colour. Large salt crystals and foam on the edges of the lake. Edges very salty like that of a salt pan.
‘Swamp’ Lake	S68˚37.345’, E078˚13.527’	8/03/2014	-1	-6	100	1.0257 / 43	28,320	Mud	Mud, boulders	Covered with approximately 1-2cm of ice.
Unnamed Lake 12	S68˚28.483’, E078˚16.363’	6/01/2015	12	11	600	1.0056 / 19		Sand, rock	Sand, rock	Thick algal mat on bottom of lake. Blizzard tails of snow leading into the lake. Some ice coverage.
Unnamed Lake 7	S68˚28.0242’, E078˚16.9309	6/01/2015	9	7	1000	1.0085 / 21		Rock, sand	Rock, sand	Thick algal mat on bottom of lake. Blizzard tails of snow leading into the lake. Two unnamed lakes 7 and 8 are joined together. Sample collected in the section joining the two lakes. Unnamed Lake 7 had some ice coverage. Unnamed Lake 8 was mostly covered in ice.

* Depth at sampling site: < 30 cm.

Figure S1│Expedition sampling of Antarctic, Vestfold Hills lakes that harboured *Candidatus* Organicella extenuata and *Euplotes* sp. AntOrgLke. **a**,The Vestfold Hills are located in East Antarctica at approximately 78° 15′ E longitude and 68° 33′ S latitude (Gibson, 1999; Cavicchioli, 2015). The ~400 km^2 region contains hundreds of water bodies, many of which were formed ~3,000–7,000 years ago after isostatic rebound of the Antarctic continent, resulting in many lakes forming with seed populations of marine microorganisms. Credit to Google Earth (Image Landsat/Copernicus; Image US Geological Survey; US Dept. of State Geographer; Data SIO, NOAA, US Navy, NGA, GEBCO). **b-h**, Organic Lake (68°27′22.2″S, 78°11′23.9″E) sampled between November 2013 and January 2015 during an overwintering expedition with samples taken representing a complete seasonal cycle. Images show sampling setup, aerial and land views of the lake and surrounds. A summary of sampling periods and depths is included (**f**). **i-l**, Other lakes sampled during the overwintering expedition, in 2014 and 2015. A summary of sampling periods and depths is included (**l**). Photo credits: Ricardo Cavicchioli, Sarah Brazendale and Alyce Hancock.

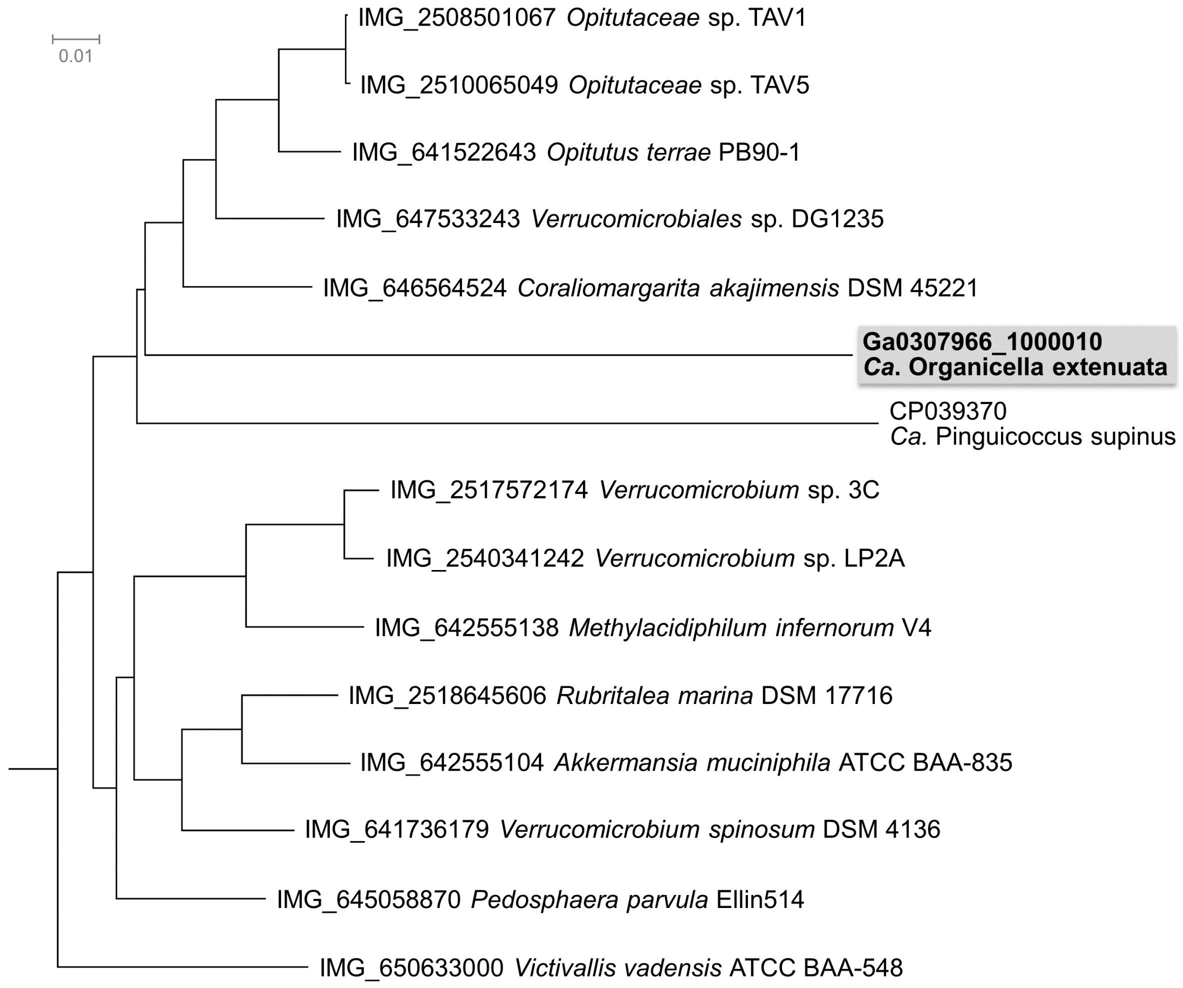

Figure S2|Phylogeny of *Candidatus* Organicella extenuata using concatenated marker genes. Phylogenetic placement of the *Ca*. Organicella MAG (Ga0307966_1000010) amongst verrucomicrobial taxa in the CheckM reference tree (Parks et al., 2014). Thirty-five of the 43 single-copy universal marker genes that the tree is based on were detected in *Ca*. Organicella, and 34 were detected in *Ca*. Pinguicoccus (33 marker genes in common). Both *Ca.* Organicella and *Ca.* Pinguicoccus have much longer branch lengths than the surrounding species, reflecting the higher rate of genomic evolution that has occurred in *Ca.* Organicella and *Ca.* Pinguicoccus compared to other Verrucomicrobia.

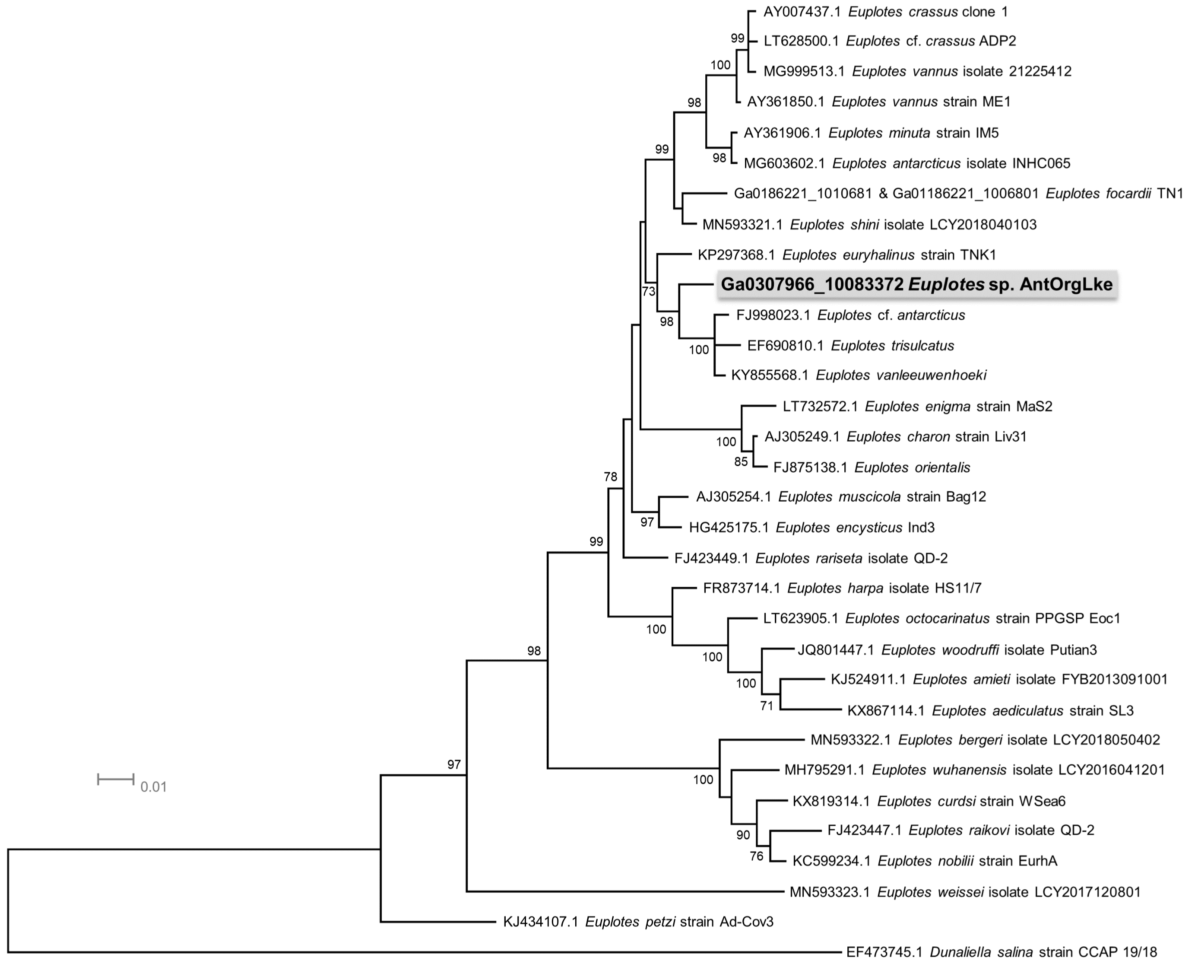

Figure S3│Phylogeny of *Euplotes* sp. AntOrgLke using 18S rRNA gene sequences. Phylogeny of *Euplotes* spp. showing placement of *Euplotes* sp. AntOrgLke as closely related to *E.* cf. *antarcticus* and *E. vanleeuwenhoeki*. The Maximum-Likelihood tree was constructed with 32 18S-rRNA nucleotide sequences, with 1748 positions remaining in the dataset after positions with less than 80% site coverage were eliminated. Bootstrap values > 70% are shown next to the nodes, and *Dunaliella salina* was used as the outgroup.

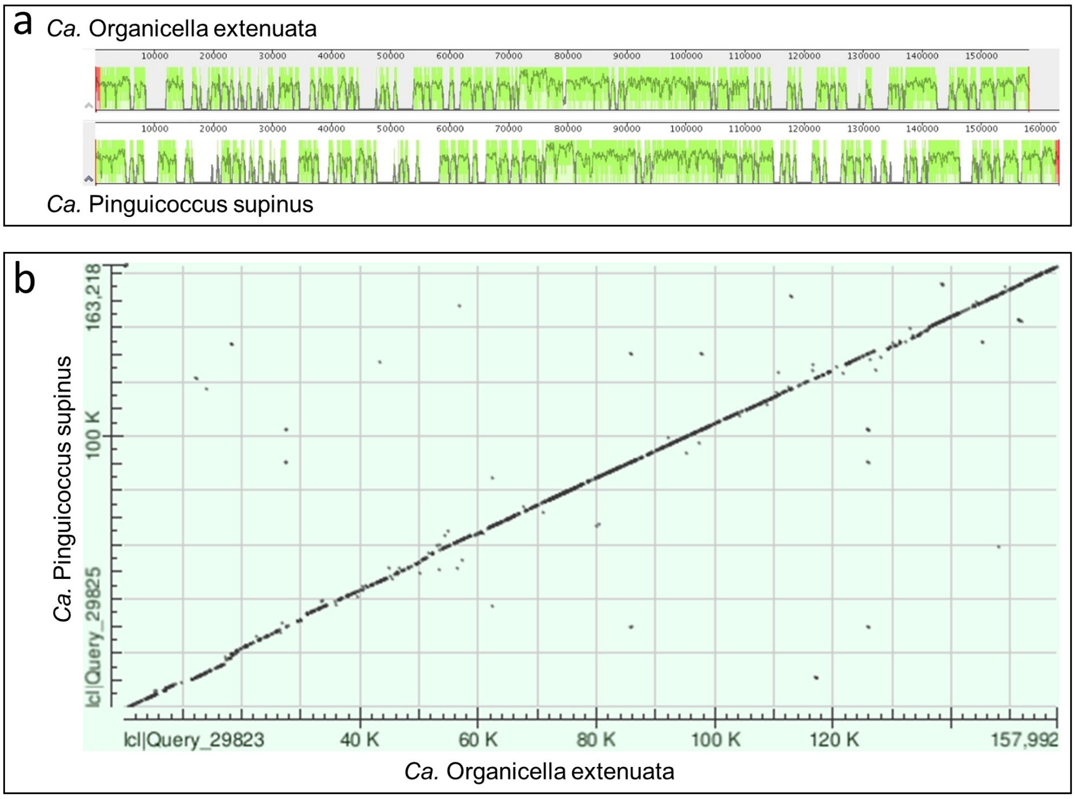

Figure S4 | Synteny of *Ca*. Organicella and *Ca*. Pinguicoccus genomes. Significant synteny between the *Ca*. Organicella and *Ca*. Pinguicoccus genomes was observed using mauve visualization (**a**) and tblastx dotplot (**b**). To align the genomes from the same starting point for ease of viewing, the circular *Ca*. Organicella MAG was split at 35600bp for both analyses.

Table S1|Characteristics of bacteria with highly reduced genomes. The host-restricted pathogen *Mycoplasma genitalium* (**) has the smallest genome size reported for a bacterium that is capable of being cultivated axenically (McCutcheon and Moran, 2012). Other than *M. genitalium*, all other bacteria listed are reported to be obligate endosymbionts. The characteristics proposed for *Candidatus* Organicella extenuata are included for comparison. Endosymbionts are grouped by host, and listed in approximate ascending order of genome size after the *Ca.* Organicella extenuata entry. The verrucomicrobial endosymbionts *Ca.* Pinguicoccus supinus, *Ca.* Xiphinematobacter americani, and *Ca.* Nucleococcus spp. are included for comparison to *Ca.* Organicella extenuata.

**Name**	**Taxonomy**	**Host**	**Benefit to host**	**Genome size (bp)**	**GC content (%)**	**Predicted CDS**	**References**
***Mycoplasma genitalium* G-37	Mollicutes, Mycoplasmatales	human (*Homo sapiens*)	none (pathogenic)	580,070	32	470	Fraser et al., 1995
***Ca.* Organicella extenuata**	**Verrucomicrobia, 'Nucleococcus cluster'**	**ciliate ('*Euplotes* sp. AntOrgLke')**	**Fe-S clusters, fatty acids**	**158,228**	**32**	**156**	**this publication**
Ca. Pinguicoccus supinus	Verrucomicrobia, 'Nucleococcus cluster'	ciliate (*Euplotes vanleeuwenhoeki*)	not stated	163,218	25	168	Serra et al., 2020
Ca. Nasuia deltocephalinicola ALF	Betaproteobacteria, Oxalobacteraceae ('BetaSymb clade')	leafhopper (*Macrosteles quadrilineatus*) (co-symbionts)	amino acids	112,091	17	137	Bennett and Moran, 2013
Ca. Sulcia muelleri ALF	Bacteroidetes, Flavobacteriales			190,733	24	190	Bennett and Moran, 2013
Ca. Tremblaya princeps PCIT	Betaproteobacteria, Burkholderiaceae	mealybug (*Planococcus citri*); nested symbiosis: *Moranella* inside *Tremblaya*	amino acids	138,927	59	121	McCutcheon and von Dohlen, 2011; Alves et al., 2013
Ca. Moranella endobia PCIT	Gammaproteobacteria, Enterobacterales			583,294	44	406	McCutcheon and von Dohlen, 2011
Ca. Hodgkinia cicadicola Dsem	Alphaproteobacteria, Rhizobiales	cicada (*Diceroprocta semicincta*) (co-symbionts)	amino acids, vitamins	143,795	58	169	McCutcheon et al., 2009
Ca. Sulcia muelleri SMDSEM	Bacteroidetes, Flavobacteriales			276,984	23	242	McCutcheon et al., 2009

Ca. Carsonella ruddii PV	Gammaproteobacteria, Oceanospirillales	gall psyllid (*Pachypsylla venusta*)	amino acids	159,662	17	182	Nakabachi et al., 2006
Ca. Tremblaya phenacola PPER	Betaproteobacteria, Burkholderiaceae	mealybug (*Phenacoccus peruvianus*)	amino acids	204,624 (198,035 + repeats 6589)	36	192	Alves et al., 2013; Gil et al., 2018
Ca. Zinderia insecticola CARI	Betaproteobacteria, Oxalobacteraceae ('BetaSymb clade')	spittlebug (*Clastoptera arizonana*) (co-symbionts)	amino acids	208,564	14	202	McCutcheon and Moran, 2010
Ca. Sulcia muelleri CARI	Bacteroidetes, Flavobacteriales			276,511	21	246	McCutcheon and Moran, 2010
Ca. Sulcia muelleri GWSS	Bacteroidetes, Flavobacteriales	sharpshooter (*Homalodisca vitripennis*) (co-symbionts)	amino acids, vitamins/ cofactors	245,530	22	227	McCutcheon and Moran, 2007
Ca. Baumannia cicadellinicola Hc	Gammaproteobacteria, Enterobacterales			686,192	33	605	Wu et al., 2006
Ca. Buchnera aphidicola sp. APS	Gammaproteobacteria, Enterobacterales	aphid (*Acyrthosiphon pisum*)	amino acids	640,681 (+ 2 small plasmids)	26	583	Shigenobu et al., 2000
Ca. Wigglesworthia glossinidia brevipalpis	Gammaproteobacteria, Enterobacterales	tstetse fly (*Glossina* spp.)	vitamins	697,742 + 5,280 plasmid	22	621	Aksoy, 1995; Akman et al., 2002
Candidatus Blochmannia floridanus	Gammaproteobacteria, Enterobacterales	carpenter ant (*Camponotus floridanus*)	amino acids, possibly fatty acids	705,557	27	583	Zientz et al., 2004; Gil et al., 2018
Ca. Xiphinematobacter americani	Verrucomicrobia	dagger nematode (*Xiphinema americanum*)	amino acids, vitamins/ cofactors	915,884	48	817	Brown et al., 2015
Ca. Nucleococcus trichonymphae, *Ca.* N. kirbyi	Verrucomicrobia, 'Nucleococcus cluster'	termite hindgut protists (*Trichonympha* spp.)	not stated	~ 1 Mb	not stated	~ 700	Sato et al., 2009; Y. Hongoh, pers. comm.

Table S3|Overview of functional capacities of *Candidatus* Organicella extenuata and *Candidatus* Pinguicoccus supinus inferred from genomic potential. Genes and proteins of *Ca.* Organicella extenuata that have no identifiable homolog in *Ca.* Pinguicoccus supinus are highlighted in red; genes and proteins of *Ca.* Pinguicoccus supinus that have no identifiable homolog in *Ca.* Organicella extenuata are highlighted in blue.

**Function**	***Ca.* Organicella extenuata**	***Ca.* Pinguicoccus supinus**
Replication	DNA gyrase subunit A (GyrA, GyrB)<br>DNA primase (DnaG)<br>replicative DNA helicase (DnaB)<br>single-stranded DNA-binding protein (Ssb)	DNA gyrase subunit A (GyrA, GyrB)<br>DNA primase (DnaG)<br>replicative DNA helicase (DnaB)<br>single-stranded DNA-binding protein (Ssb)
Transcription	RNAP (RpoA, RpoB, RpoC)<br>sigma factor (RpoD)<br>transcription factor NusG	RNAP (RpoA, RpoB, RpoC)<br>sigma factor (RpoD)<br>transcription factor NusG<br>transcriptional regulator (RNAP σ^{54} interaction domain)
DNA repair	RecA	RecA
RNA degradation	ribonuclease E (Rne)	-
tRNA modification	tRNA wobble uridine modification enzymes: aminomethyl or carboxymethylaminomethyl incorporation (MnmE, MnmG); thiouridylation (MnmA)<br>tRNA N6-adenosine threonylcarbamoyltransferase (TsaD)<br>tRNA(Ile)-lysidine synthase (TilS)	tRNA wobble uridine modification enzymes: aminomethyl or carboxymethylaminomethyl incorporation (MnmE, MnmG); thiouridylation (MnmA)<br>tRNA N6-adenosine threonylcarbamoyltransferase (TsaD)<br>tRNA(Ile)-lysidine synthase (TilS)
Translation	initiation factors (InfA, InfB)<br>elongation factors (Tsf, FusA, LepA)<br>peptide chain release factor (PrfA)<br>ribosomal RNA genes (23S, 16S, 5S)<br>30S ribosomal proteins (RpsA, RpsB, RpsC, RpsD, RpsE, RpsG, RpsH, RpsI, RpsJ, RpsK, RpsL, RpsM, RpsN, RpsO, RpsP, RpsQ, RpsS, RpsT, RpsU)<br>50S ribosomal proteins (RplB, RplC, RplD, RplE, RplF, RplL, RplJ, RplK, RplM, RplN, RplO, RplP, RplQ, RplR, RplS, RplT, RplU, RplV, RplW, RplX, RplY, RpmA, RpmB, RpmE, RpmH, RpmI)<br>ribosome-recycling factor (Frr)<br>aminoacyl tRNA synthetases for Met, Leu, Ile, Val, Lys, Gly, Ser, Cys, Arg, Tyr, Ala, Phe, Glu; also aspartyl/glutamyl-tRNA amidotransferase<br>tRNAs for Ala, Arg(2), Asn, Asp, Cys, Gln, Glu, Gly(2), His, Ile, Leu(4), Lys(2), Met(3), Phe, Pro, Ser(4), Thr(3), Trp, Tyr, Val(2)	initiation factors (InfB, InfC)<br>elongation factors (Tsf, FusA, LepA (2))<br>peptide chain release factor (PrfA)<br>ribosomal RNA genes (23S, 16S, 5S)<br>30S ribosomal proteins (RpsA, RpsB, RpsC, RpsD, RpsE, RpsG, RpsH, RpsI, RpsJ, RpsK, RpsL, RpsM, RpsN, RpsO, RpsP, RpsR, RpsS, RpsT, RpsU)<br>50S ribosomal proteins (RplB, RplC, RplD, RplE, RplF, RplL, RplI, RplJ, RplK, RplM, RplN, RplO, RplP, RplQ, RplR, RplT, RplU, RplV, RplY, RpmA, RpmB, RpmE, RpmH, RpmI, RpmJ)<br>ribosome-recycling factor (Frr)<br>aminoacyl tRNA synthetases for Met, Leu, Ile, Val, Lys, Ser, Cys, Arg, Tyr, Ala, Phe, Trp; also aspartyl/glutamyl-tRNA amidotransferase<br>tRNAs for Ala, Arg(2), Asn, Asp, Cys, Gln, Glu, Gly(2), His, Ile, Leu(4), Lys(2), Met(3), Phe, Pro, Ser(4), Thr(3), Trp, Tyr, Val(2)

	methionyl-tRNA formyltransferase (Fmt)<br>peptide deformylase (Def)	
Protein-folding or degradation	chaperone proteins (GrpE, DnaJ, DnaK)<br>chaperonins (GroEL, GroES)<br>Clp protease (ClpP1, ClpP2, ClpX)	chaperone proteins (GrpE, DnaJ, DnaK)<br>chaperonins (GroEL, GroES)<br>Clp protease (ClpP1, ClpP2, ClpX)
Pyruvate metabolism	pyruvate dehydrogenase E1 (PdhA, PdhB) (two sets), dihydrolipoyllysine-residue acetyltransferase E2 component (PdhC), dihydrolipoyl dehydrogenase E3 component (LpdA)	pyruvate dehydrogenase E1 (PdhA, PdhB) (two sets), dihydrolipoyllysine-residue acetyltransferase E2 component (PdhC), dihydrolipoyl dehydrogenase E3 component (LpdA)
Fatty acid metabolism	acetyl-coenzyme A carboxylase complex (AccA, AccC, AccB, AccD), acyl carrier protein (AcpP), malonyl-CoA-ACP-transacylase (FabD), 3-oxoacyl-ACP synthase 1 (FabB), 3-oxoacyl-ACP reductase (FabG), 3-hydroxyacyl-ACP-dehydratase (FabZ), enoyl-ACP reductase (FabV), 3-oxoacyl-ACP synthase 2 (FabF)	acetyl-coenzyme A carboxylase complex (AccA, AccC, AccB, AccD), acyl carrier protein (AcpP), malonyl-CoA-ACP-transacylase (FabD), 3-oxoacyl-ACP synthase 1 (FabB), 3-oxoacyl-ACP reductase (FabG), 3-hydroxyacyl-ACP-dehydratase (FabZ), enoyl-ACP reductase (FabV), 3-oxoacyl-ACP synthase 2 (FabF), CDP-diacylglycerol--glycerol-3-phosphate 3-phosphatidyltransferase (PgsA)
Heptose metabolism	transketolase (Tkl), phosphoheptose isomerase (GmhA), sugar-phosphate kinase (HddA), sugar-phosphate guanylyltransferase (HddC)	transketolase (Tkl), phosphoheptose isomerase (GmhA), sugar-phosphate kinase (HddA), sugar-phosphate guanylyltransferase (HddC)
Hexose metabolism	nucleotide-sugar dehydratase/epimerase (GalE/Agl12), UDP-glucose dehydrogenase (Udg), UDP-glucuronate 5'-epimerase (LpsL), GDP-4-keto-6-deoxy-D-mannose 3-dehydratase (ColD), GDP-L-colitose synthase (ColC)/GDP-L-fucose synthase (Fcl)	nucleotide-sugar dehydratase/epimerase (GalE/Agl12), UDP-glucose dehydrogenase (Udg), UDP-glucuronate 5'-epimerase (LpsL), GDP-4-keto-6-deoxy-D-mannose 3-dehydratase (ColD), GDP-L-colitose synthase (ColC)/GDP-L-fucose synthase (Fcl)
Glycosyl transfers	glycosyltransferases (eight) of uncertain specificities	glycosyltransferases (eight) of uncertain specificities
FeS clusters	FeS cluster assembly proteins (SufC, SufB, SufD, SufU, SufT), cysteine desulfurase (SufS), ferredoxin	FeS cluster assembly proteins (SufC, SufB, SufD, SufU, SufT), cysteine desulfurase (SufS), ferredoxin
Redox balance	-	thioredoxin (TrxA), thioredoxin reductase (TrxB)
Amino acid metabolism	-	glutamate dehydrogenase (NADP)

Table S8 | Amino acid identity between *Euplotes* species.

	Euplotes sp. AntOrgLke	*E. octocarinatus*	*E. focardii*	*E. crassus*	*E. harpa*
E. octocarinatus	57				
E. focardii	55	53			
E. crassus	57	53	61		
E. harpa	56	59	54	54	
E. vannus	53	46	54	91	49

Proteins were predicted from available genome data. *Euplotes* sp. AntOrgLake (15,328 proteins); *E. octocarinatus* (29,076 proteins) obtained from http://ciliates.ihb.ac.cn/database/home/#eo; *E. focardii* TN1 (12,634 proteins), *E. crassus* CT5 (12,729 proteins) and *E. harpa* FSP1.4 (19,386 proteins) predicted from the Marine Microbial Eukaryote Meta/transcriptome sequencing project (MMETSP) was accessed from http://img.jgi.doe.gov; *E. vannus* (43,338 proteins) obtained from http://evan.ciliate.org. Two-way AAI is shown as a percentage.

Supplementary References

Akman, L., Yamashita, A., Watanabe, H., Oshima, K., Shiba, T., Hattori, M., and Aksoy, S. 2002. Genome sequence of the endocellular obligate symbiont of tsetse flies, Wigglesworthia glossinidia Nat. Genet. 2002. 32: 402-407.

Aksoy, S. 1995 *Wigglesworthia* gen. nov. and *Wigglesworthia glossinidia* sp. nov., taxa consisting of the mycetocyte-associated, primary endosymbionts of tsetse flies. Int J Syst Bacteriol. 45:848-851.

Alves JM, Serrano MG, Maia da Silva F, Voegtly LJ, Matveyev AV, Teixeira MM, Camargo EP, Buck GA. (2013). Genome evolution and phylogenomic analysis of *Candidatus* Kinetoplastibacterium, the betaproteobacterial endosymbionts of *Strigomonas* and *Angomonas*. Genome Biol Evol. 5:338-350

Armengod, M.E., Moukadiri, I., Prado, S., Ruiz-Partida, R., Benitez-Paez, A., Villarroya, M., Lomas, R., Garzon, M.J., Martinez-Zamora, A., Meseguer, S., Navarro-González C. 2012. Enzymology of tRNA modification in the bacterial MnmEG pathway. Biochimie 94: 1510-1520

Bennett GM, Moran NA. 2013. Small, smaller, smallest: the origins and evolution of ancient dual symbioses in a phloem-feeding insect. Genome Biol. Evol. 5:1675–1688

Broach B, Gu X, Bar-Peled M (2012) Biosynthesis of UDP-glucuronic acid and UDP-galacturonic acid in *Bacillus cereus* subsp. cytotoxis NVH 391-98. FEBS J 279:100-12

Brown AM, Howe DK, Wasala SK, Peetz AB, Zasada IA, Denver DR. (2015) Comparative genomics of a plant-parasitic nematode endosymbiont suggest a role in nutritional symbiosis. Genome Biol Evol. 7: 2727-2746.

Cavicchioli, R. Microbial ecology of Antarctic aquatic systems. Nat. Rev. Microbiol. 13, 691–706 (2015).

Cook PD, Holden HM (2008) GDP-4-keto-6-deoxy-D-mannose 3-dehydratase, accommodating a sugar substrate in the active site. J Biol Chem 283:4295-4303.

Deutsch,C., El Yacoubi,B., de Crecy-Lagard,V. and Iwata-Reuyl,D. (2012) Biosynthesis of threonylcarbamoyl adenosine(t^6A), a universal tRNA nucleoside. J Biol. Chem. 287: 13666-13673.

Fraser CM, Gocayne JD, White O, Adams MD, Clayton RA, Fleischmann RD, Bult CJ, Kerlavage AR, Sutton G, Kelley JM, Fritchman RD, Weidman JF, Small KV, Sandusky M, Fuhrmann J, Nguyen D, Utterback TR, Saudek DM, Phillips CA, Merrick JM, Tomb JF, Dougherty BA, Bott KF, Hu PC, Lucier TS, Peterson SN, Smith HO, Hutchison CA 3rd, Venter JC. (1995) The minimal gene complement of *Mycoplasma genitalium*. Science. 270:397-403.

Gibson JAE. (1999). The meromictic lakes and stratified marine basins of the Vestfold Hills, East Antarctica. Antarct Sci 11: 175-192.

Gil R, Vargas-Chavez C, López-Madrigal S, Santos-García D, Latorre A, Moya A. (2018) *Tremblaya phenacola* PPER: an evolutionary beta-gammaproteobacterium collage. ISME J. 12:124-135.

Kambampati R and Lauhon CT (2003). MnmA and IscS are required for in vitro 2- thiouridine biosynthesis in *Escherichia coli* Biochemistry 42: 1109-1117

Kaminski L, Eichler J (2014) *Haloferax volcanii* N-Glycosylation: Delineating the pathway of dTDP-rhamnose biosynthesis. PLoS ONE 9: e97441

Kaminski L., Guan Z., Yurist-Doutsch S., Eichler J. (2013). Two distinct N-glycosylation pathways process the *Haloferax volcanii* S-layer glycoprotein upon changes in environmental salinity. MBio 4:E00716-E00716

Karlyshev AV, Champion OL, Churcher C, Brisson JR, Jarrell HC, Gilbert M, Brochu D, St Michael F, Li J, Wakarchuk WW, Goodhead I, Sanders M, Stevens K, White B, Parkhill J, Wren BW, Szymanski CM. (2005) Analysis of *Campylobacter jejuni* capsular loci reveals multiple mechanisms for the generation of structural diversity and the ability to form complex heptoses. Mol. Microbiol. 55:90-103

Kereszt, A., E. Kiss, B. L. Reuhs, R. W. Carlson, K. A. Kondorosi, and P.Putnoky.1998. Novel *rkp* gene clusters of *Sinorhizobium meliloti* involved incapsular polysaccharide production and invasion of the symbiotic nodule: the *rkp* gene encodes a UDP-glucose dehydrogenase. J. Bacteriol. 180: 5426-5431

Kneidinger, B., C. L. Marolda, M. Graninger, A. Zamyatina, F. McArthur, P. Kosma, M. A. Valvano, and P. Messner. 2002. Biosynthesis pathway of ADP-L-*glycero*-β-D-*manno*-heptose in *Escherichia coli*. J. Bacteriol. 184:363-369

Kneidinger, B., M. Graninger, M. Puchberger, P. Kosma, and P. Messner. 2001. Biosynthesis of nucleotide-activated D-*glycero*-D-*manno*-heptose. J. Biol. Chem. 276: 20935–20944

McAllister KA, Peery RB, Zhao G. (2006) Acyl carrier protein synthases from Gram-negative, Gram-positive, and atypical bacterial species: Biochemical and structural properties and physiological implications. J Bacteriol. 188:4737-4748.

McCutcheon JP, McDonald BR, Moran NA. (2009). Origin of an alternative genetic code in the extremely small and GC-rich genome of a bacterial symbiont. PLoS Genet. 5:e1000565

McCutcheon JP, Moran NA. (2007) Parallel genomic evolution and metabolic interdependence in an ancient symbiosis. Proc Natl Acad Sci U S A. 104:19392-19397.

McCutcheon JP, Moran NA. (2010) Functional convergence in reduced genomes of bacterial symbionts spanning 200 My of evolution. Genome Biol Evol. 2:708-718.

McCutcheon JP, Moran NA. (2012) Extreme genome reduction in symbiotic bacteria. Nat Rev Microbiol. 10:13-26.

McCutcheon JP, von Dohlen CD. (2011) An interdependent metabolic patchwork in the nested symbiosis of mealybugs. Curr Biol. 2011 21:1366-1372.

Moukadiri I, Garzón MJ, Björk GR, Armengod ME. The output of the tRNA modification pathways controlled by the *Escherichia coli* MnmEG and MnmC enzymes depends on the growth conditions and the tRNA species. Nucleic Acids Res. 2014 ;42: 2602-2623.

Nakabachi A, Yamashita A, Toh H, Ishikawa H, Dunbar HE, Moran NA, Hattori M. (2006) The 160-kilobase genome of the bacterial endosymbiont *Carsonella*. Science. 314:267.

Parks DH, Imelfort M, Skennerton CT, Hugenholtz P, Tyson GW. 2014. Assessing the quality of microbial genomes recovered from isolates, single cells, and metagenomes. Genome Research, 25: 1043-1055.

Parsons JB, Rock CO (2013). Bacterial lipids: Metabolism and membrane homeostasis. Progress in Lipid Research 52: 249–276

Samuel G. and Reeves P. 2003 Biosynthesis of O-antigens: genes and pathways involved in nucleotide sugar precursor synthesis and O-antigen assembly. Carbohydrate Res 338: 2503-2519

Sato T, Hongoh Y, Noda S, Hattori S, Ui S, Ohkuma M. (2009). *Candidatus* Desulfovibrio trichonymphae, a novel intracellular symbiont of the flagellate *Trichonympha agilis* in termite gut. Environ Microbiol 11: 1007–1015.

Seemann T (2014) Prokka: rapid prokaryotic genome annotation. Bioinformatics 30:2068-2069.

Serra, V., Gammuto, L., Nitla, V. *et al.* Morphology, ultrastructure, genomics, and phylogeny of *Euplotes vanleeuwenhoeki* sp. nov. and its ultra-reduced endosymbiont "*Candidatus* Pinguicoccus supinus" sp. nov.. *Sci Rep* **10,** 20311 (2020).

Shigenobu S, Watanabe H, Hattori M, Sakaki Y, Ishikawa H. (2000) Genome sequence of the endocellular bacterial symbiont of aphids *Buchnera* sp. APS. Nature. 407:81-86.

Soma A, Ikeuchi Y, Kanemasa S, Kobayashi K, Ogasawara N, Ote T, Kato J, Watanabe K, Sekine Y, Suzuki T (2003). An RNA-modifying enzyme that governs both the codon and amino acid specificities of isoleucine tRNA. Mol. Cell 12: 689-698

Taylor PL, Blakely KM, de Leon GP, Walker JR, McArthur F, Evdokimova E, Zhang K, Valvano MA, Wright GD, Junop MS. Structure and function of sedoheptulose-7-phosphate isomerase, a critical enzyme for lipopolysaccharide biosynthesis and a target for antibiotic adjuvants. J Biol Chem. 2008 283:2835-2845.

Wan, L.C.K., Mao, D.Y.L., Neculai, D., Strecker, J., Chiovitti, D., Kurinov, I., Poda, G., Thevakumaran, N., Yuan, F., Szilard, R.K., Lissina, E., Nislow, C., Caudy, A.A., Durocher, D., Sicheri, F. (2013) Reconstitution and characterization of eukaryotic N6-threonylcarbamoylation of tRNA using a minimal enzyme system. Nucleic Acids Res. 41: 6332-6346

Wu D., Daugherty S.C., Van Aken S.E., Pai G.H., Watkins K.L., Khouri H., Tallon L.J., Zaborsky J.M., Dunbar H.E., Tran P.L., Moran N.A., Eisen J.A. (2006) Metabolic complementarity and genomics of the dual bacterial symbiosis of sharpshooters. PLoS Biol. 4:1079-1092

Zientz E, Dandekar T, Gross R. (2004) Metabolic interdependence of obligate intracellular bacteria and their insect hosts. Microbiol Mol Biol Rev. 68:745-770.
